# Dichloroacetyl Amides of 3,5-Bis(benzylidene)-4-piperidones Displaying Greater Toxicity to Neoplasms than to Non-Malignant Cells

**DOI:** 10.3390/medicines9060035

**Published:** 2022-06-08

**Authors:** Mohammad Hossain, Praveen K. Roayapalley, Hiroshi Sakagami, Keitaro Satoh, Kenjiro Bandow, Umashankar Das, Jonathan R. Dimmock

**Affiliations:** 1 School of Sciences, Indiana University Kokomo, Kokomo, IN 46904, USA; mohoss@iu.edu; 2 Drug Discovery and Development Research Cluster, University of Saskatchewan, Saskatoon, SK S7N 5E5, Canada; umashankar.usask@gmail.com (U.D.); jr.dimmock@usask.ca (J.R.D.); 3 Meikai University School of Dentistry, Sakado 350-0283, Japan; sakagami@dent.meikai.ac.jp (H.S.); k-satoh@dent.asahi-u.ac.jp (K.S.); kbando@dent.meikai.ac.jp (K.B.)

**Keywords:** unsaturated ketones, cytotoxicity, dichloroacetic acid, tumor-selective toxicity, structure-activity relationships

## Abstract

A series of 3,5-bis(benzylidene)-1-dichloroacetyl-4-piperidones **1a**–**l** was evaluated against Ca9-22, HSC-2, HSC-3, and HSC-4 squamous cell carcinomas. Virtually all of the compounds displayed potent cytotoxicity, with 83% of the CC_50_ values being submicromolar and several CC_50_ values being in the double digit nanomolar range. The compounds were appreciably less toxic to human HGF, HPLF, and HPC non-malignant cells, which led to some noteworthy selectivity index (SI) figures. From these studies, **1d**,**g**,**k** emerged as the lead molecules in terms of their potencies and SI values. A Quantitative Structure-Activity Relationship (QSAR) study revealed that cytotoxic potencies and potency–selectivity expression figures increased when the magnitude of the sigma values in the aryl rings was elevated. The modes of action of the representative cytotoxins in Ca9-22 cells were found to include G2/M arrest and stimulation of the cells to undergo mitosis and cause poly(ADP-ribose) polymerase (PARP) and procaspase 3 cleavage.

## 1. Introduction

A current emphasis in medicinal chemistry is the creation of hybrid molecules formed from two or more bioactive compounds [1,2,3]. The value of these molecules includes the following considerations. First, hybrid compounds may interact at a greater number of binding sites than drugs, which act principally at one binding site. Second, the development of drug resistance may be reduced or eliminated in drug hybrids, which have multiple sites of action.

Glycolysis is enhanced in certain tumors, and this process may involve the use of the pyruvate dehydrogenase complex (PDC). Phosphorylation of the PDC is catalyzed by four isoforms of pyruvate dehydrogenase kinase, referred to as PDK 1–4, which are present in a number of human tumors [4]. Dichloroacetic acid (DCA) is an inhibitor of PDK 1 [5], and this inhibition is considered to have a detrimental effect on tumor growth.

There are growing interests in the development of new approaches focused on creating DCA derivatives, including enhanced tolerability, safety, and the ability to cross cellular membranes, thereby facilitating drug uptake. During the last decade, many DCA derivatives of natural products [6,7,8,9], synthetic organics [10,11], and inorganic compounds [12,13] have been synthesized and evaluated for anticancer activity. For example, amides and esters of DCA and structurally related naturally occurring compounds such as albiziabioside A [6], doxorubicin [7], phenstatin [8], and honokiol [9] have been investigated in vitro against various cancer cells. The dichloroacetamide of the triterpenoid saponin albiziabioside A has been reported to show selective cytotoxicity against PDK-medium and PDK-high expressed human cancer cells [6]. The DCA-albiziabioside A amide displayed superior cytotoxicity compared with albiziabioside A and DCA alone against the cancer cells HCT116, A375, and 4T1, showing the best cytotoxicity against the PDK-high expressed human breast cancer cells MCF-7, possibly more efficiently than PDK inhibition capacity. The amide can also inhibit both primary and distal tumor progression in a dual-4T1 tumor model in female BALB/c mice. It was reported that the dichloroacetamide derivative of doxorubicin can self-assemble into nanoparticles with a small amount of liquid polymer, which exhibits a high loading content with greatly decreased side effects caused by the excipient [7]. The nanoparticles did not exhibit observable systemic toxicity and had a high maximum tolerated dose of the doxorubicin-DCA derivative, which was 15-fold higher than that of free doxorubicin. It also showed good in vivo tumor-targeting capability and enhanced antitumor efficacy in a murine melanoma model.

A series of dual-targeted compounds have been synthesized by combining anti-tubulin benzophenones and benzothiophenones derived from phenstatin, a known potent tubulin polymerization inhibitor, with mono-, di- and tri-chloroacetate groups targeting PDK1 [8]. Some of these synthesized compounds can act as inhibitors of PDK1, and some are dual inhibitors of both tubulin and PDK1.

Extensive QSAR (quantitative structure-activity relationships) and molecular docking studies have been conducted on a series of *N*-aryldichloroacetamide and aryl dichloroacetate derivatives using PDKs isoenzymes, which suggested that a number of hydrogen bond acceptor interactions exist between the oxygen of amidic carbonyl group and different amino acid residues in various PDKs [14]. Molecular docking studies using honokiol bis-dichloroacetate and TNF receptor-associated protein 1 (TRAP1) showed important contacts between the ligand and the protein residues in the allosteric pocket, and as a result, honokiol bis-dichloroacetate could act as a selective allosteric inhibitor of the mitochondrial chaperone TRAP1 [15].

Thus, the decision was made to incorporate the dichloroacetyl group into the hybrid molecules. However, DCA is considered to be a weak anticancer agent [2], and it was considered judicious to attach the dichloroacetyl group to a series of potent cytotoxins. Previously, we have shown that a number of 3,5-bis(benzylidene)-4-piperidones have noteworthy cytotoxic activity [16]. These observations led to the decision to prepare the dichloroacetamides **1a**–**l**. A preliminary communication revealed that most of these compounds demonstrated significant cytotoxic potencies in the region of 10^−6^ and 10^−7^ M towards human HCT 116 colon cancer cells and in general are far less toxic towards human non-malignant CRL1790 colon cells [2].

The aims of our investigation were as follows. First, we sought to find out if the compounds in series **1** are cytotoxic to other malignant cell lines. Second, an important feature of this series of compounds is whether greater toxicity to neoplasms than to non-malignant cells is demonstrated. A third line of inquiry was directed to finding some of the modes of action of representative compounds. The results from these investigations should enable a decision to be reached as to the wisdom of expanding the project. 

## 2. Experimental Methods

### 2.1. Synthesis of Compounds

The unsaturated ketones **1a**–**l**, **2a**,**b** were prepared by a literature procedure [2]. Copies of the ^1^H and ^13^C NMR spectra as well as the mass spectra of the compounds in series **1** and **2** are presented in the Supplemental Section of this report. ^1^H and ^13^C NMR spectra were determined in either CDCl_3_ or DMSO-*d*_6_ using a Bruker Avance III 500 MHz NMR spectrometer (Chicago, IL, USA), while mass spectra were generated using a JEOL JMS-T100GCv AccuTOF-GCv4G Mass Spectrometer (Peabody, MA, USA). 

### 2.2. Cytotoxicity Assays

The target cells used in our study were human oral squamous cell carcinoma cell lines Ca9-22 (purchased from RIKEN Cell Bank, Tukuba, RCB-1976), HSC-2 (RCB1945), HSC-3 (RCB1975), HSC-4 (RCB1902), and three human normal oral cells, gingival fibroblast (HGF), periodontal ligament fibroblast (HPLF), and pulp cells (HPC) [established from the first premolar extracted tooth in the lower jaw (because of dysfunctional position or orthodontic treatment) and periodontal tissues of a twelve-year-old girl, according to the guideline of the Institutional Board of Meikai University Ethics Committee (No. A0808)], after obtaining informed consent from the patients [17]. These cells were incubated for 48 h with the indicated concentrations of test samples or reference compounds sodium dichloroacetate (purchased from Sigma-Aldrich Chemical Company, Saint Louis, MO, USA), 5-fluorouracil (5-FU) from Kyowa (Tokyo, Japan), and doxorubicin (DXR) (St. Louis, MO, USA) and vehicle (DMSO) (0.008, 0.016, 0.031, 0.063, 0.125, 0.25, 0.5, and 1%) in Dulbecco’s Modified Eagle Medium (DMEM) media, which was supplemented with 10% heat-inactivated fetal bovine serum [18]. Cell viability was determined by the MTT method [18]. Cytotoxicity caused by the vehicle (DMSO) was subtracted.

### 2.3. Calculation of Selectivity Index (SI)

SI was calculated by dividing the average CC_50_ value (concentration of the compound to kill 50% of the cells) of the compound towards HGF, HPLF, and HPC cells by the CC_50_ value of the compound against a specific neoplastic cell line.

### 2.4. Calculation of Potency-Selectivity Expression (PSE)

PSE reflects both the potencies and differences in cytotoxicity to neoplasms and non-malignant cells. This value was the product of the reciprocal of the average CC_50_ value of the compounds towards Ca9-22, HSC-2, HSC-3, and HSC-4 cells and the average SI value × 100. 

### 2.5. Cell Cycle Assay

The evaluation of varying quantities of **1d**,**e**,**k** on the cell cycle in Ca9-22 cells was undertaken by a literature method [19]. In brief, Ca9-22 cells were treated for 20 h without (control) or with the indicated concentrations of the test samples. To neglect the cytotoxicity effect of DMSO, all samples contained 0.1% DMSO. Cells (approximately 10^6^ cells) were harvested, fixed for 1 h on ice with 1% paraformaldehyde, washed twice by phosphate-buffered saline, treated for 30 min at 37 °C with 0.2 mg/mL RNase A (ribonuclease A), stained for 15 min at room temperature with 0.01% propidium iodide in the presence of 0.01% NP-40 (nonyl phenoxypolyethoxyethanol) to prevent cell aggregation, filtered through Falcon^®^ cell strainers (pore size: 40 µm) (Corning, NY, USA), subjected to cell sorting (SH800 Series; SONY Imaging Products and Solutions Inc., Kanagawa, Japan), and then analyzed with Cell Sorter Software version 2.1.2. (SONY Imaging Products and Solutions Inc., Kanagawa, Japan).

### 2.6. PARP and Procaspase 3 Cleavage

The effect of 20 h incubation of different concentrations of **1k** and **2a** with Ca9-22 cells on the cleavage of PARP and procaspase 3 cleavage was undertaken using a reported procedure [20]. In brief, the control and treated Ca9-22 cells at near confluent phase were collected and lysed, and protein samples of cell lysates (15 µg) were applied to SDS-polyacrylamide gel electrophoresis. After electrophoresis, the separated proteins were transferred onto a PVDF (polyvinylidene fluoride) filter. The blots were treated in skim milk and then probed for 120 min with a primary antibody cocktail (1:250) from an Apoptosis Western Blot Cocktail kit (purchased from Abcam, Cambridge, UK). The blots were washed and probed with horseradish peroxidase conjugated secondary antibody cocktail (1:100). Immunoreactivities were determined using Amer-sham ECL Select. Images were acquired using ChemiDoc MP System and Image Lab 4.1 software (Bio-Rad Laboratories, Hercules, CA, USA).

### 2.7. Statistical Treatment

Statistical analyses were performed using the SPSS 23.0 (statistical package for social sciences) software (IBM, Armonk, NY, USA). Experimental data are presented as the mean ± standard deviation (SD) of triplicate determinations. The significance of values was examined by one-way analysis of variance (ANOVA) and the appropriate Dunnett’s post-test. A value of * *p* < 0.05 was considered to indicate statistically significant differences.

## 3. Results

**Figure 1:**
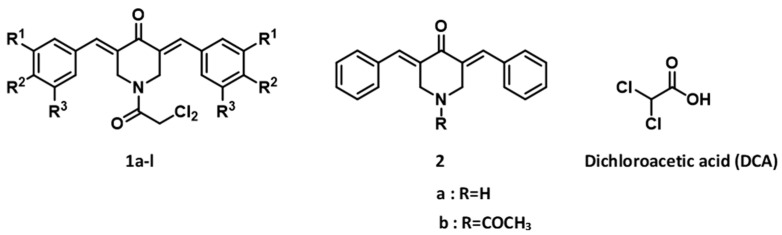
The structures of the compounds in series **1**, **2** and dichloroacetic acid (DCA). The aryl substituents in series **1** are indicated in Table 1.

The synthesis of series **1** and **2** followed a literature procedure [2]. Various aryl aldehydes were reacted with 4-piperidone to produce the corresponding 3,5-bis(benzylidene)-4-piperidones. Acylation of these intermediate unsaturated ketones with dichloroacetyl chloride gave rise to the desired products **1a**–**l**. Acylation of 3,5-bis(benzylidene)-4-piperidone **2a** with acetyl chloride led to the formation of **2b**. The structures of the compounds in series **1** and **2** are portrayed in Figure 1.

**Figure 2:**
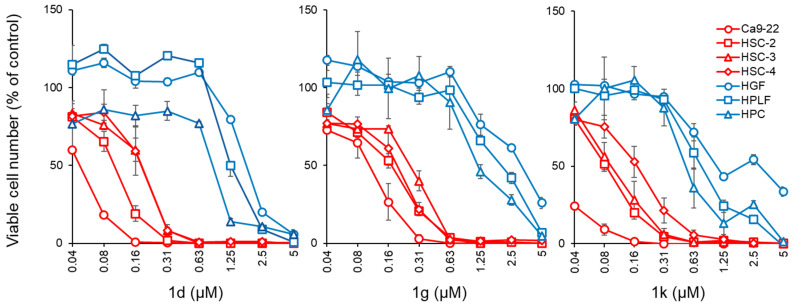
Kinetics of cytotoxicity induction by **1d**, **1g,** and **1k** on human oral squamous cell lines (Ca9-22, HSC-2, HSC-3, and HSC-4) and human normal oral cells (HGF, HPLF, and HPC). These cells were incubated for 48 h with the indicated concentrations of **1d**, **1g,** and **1k**, and the viable cell number was determined by MTT [3-(4,5-dimethylthiazol-2-yl)-2,5-diphenyltetrazolium bromide] methods. Each value represents the mean ± S.D. (standard deviation) of triplicate assays.

**Figure 3:**
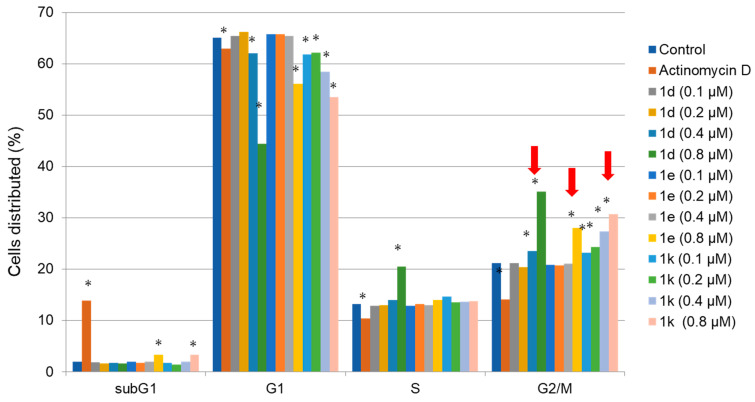
The effect of **1d**,**e**,**k** on the cell cycle in Ca9-22 cells. Cells were treated without (control) or with 1 μM actinomycin D (AD) or the indicated concentrations of **1d**, **1e,** and **1k** in the presence of the DMSO vehicle (0.1%). Experimental data are presented as the mean ± standard deviation (SD) of triplicate determinations. The significance of values was examined by one-way analysis of variance (ANOVA) and appropriate Dunnett’s post-test. A value of * *p* < 0.05 was considered to indicate statistically significant differences. The red arrows indicate the highest value of G2/M phase cells in **1d**, **1e** and **1k**.

**Figure 4:**
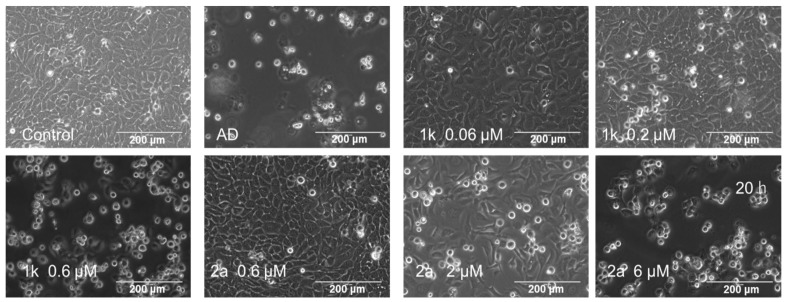
Increased mitosis in Ca9-22 cells after treatment with **1k**, **2a,** and actinomycin D(AD). Cells were treated without (control) or with 1 μM actinomycin D or the indicated concentrations of **1k** and **2a** in the presence of the DMSO vehicle (0.1%).

**Figure 5:**
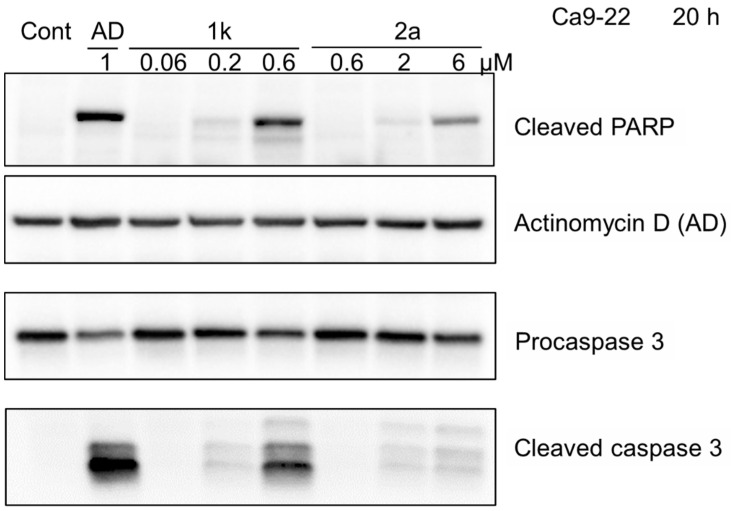
Cleavage of PARP and procaspase 3 in Ca9-22 cells by **1k**, **2a,** and actinomycin D(AD). Cells were treated without (control) or with 1 μM actinomycin D or the indicated concentrations of **1k** and **2a** in the presence of the DMSO vehicle (0.1%).

The compounds in series **1** and **2** were evaluated against human Ca9-22, HSC-2, HSC-3, and HSC-4 squamous cell carcinomas, and the results are portrayed in Table 1. In addition, these conjugated unsaturated ketones were screened against non-malignant human gingival fibroblasts (HGF), human periodontal ligament fibroblasts (HPLF), and human pulp cells (HPC). These biodata are presented in Table 2. Linear and semilogarithmic plots were made between the Hammett sigma (σ), Hansch pi (π), and molar refractivity (MR) constants of the aryl substituents and the average CC_50_ values, the average selectivity index (SI) figures, and the potency–selectivity expression (PSE) figures. The dose-response curve of three represented compounds (**1d**,**g**,**k**) against these 7 cells (four malignant and three non-malignant cells) is shown in Figure 2. Several mode of action studies of representative compounds in Ca9-22 cells were conducted. The effect of **1d**,**e**,**k** on the cell cycle is presented in Figure 3. The enones **1k** and **2a** induced mitotic accumulation in Ca9-22 cells (Figure 4), while the ability of these two compounds to cleave PARP [poly(ADP-ribose)polymerase] and procaspase-3 is portrayed in Figure 5.

## 4. Discussion

The evaluation of **1a**–**l** and **2a,b** towards Ca9-22, HSC-2, HSC-3, and HSC-4 was considered initially. The squamous cell carcinomas arising from the oral mucosal epithelium can be aggressive [21]. Hence, the discovery of novel compounds to treat this type of cancer assumes some importance. The biodata generated reveal that the dichloroacetamides in series **1** are highly potent cytotoxins in general. No less than 83% of the CC_50_ values of **1a**–**l** are submicromolar. If the outlier **1l** is removed from consideration, the figure then rises to 91%. In addition, one should note the double-digit nanomolar CC_50_ values of **1c**–**e**,**k** towards Ca9-22 cells derived from gingiva and of **1e,k** to HSC-2 carcinomas from tongue. The most potent compounds (average CC_50_ value in parentheses) are **1d** (0.13), **1e** (0.12), **1g** (0.19), and **1k** (0.10). These compounds showed potent cytotoxicity, killing all cancer cells rather than having a cytostatic effect (Figure 2). In general, **1a** had higher CC_50_ values than **2a,** but **1a** possessed lower CC_50_ figures than **2b**. Thus, considering **1a**, **2a**, and **b**, in general, *N*-acylation lowers potency.

The next question to resolve was how the cytotoxic potencies of these compounds in series **1** and **2** compared with clinically used anticancer agents. Sodium dichloroacetate had little or no efficacy in inhibiting the growth of Ca9-22, HSC-2, HSC-3, and HSC-4 cells. On the other hand, 5-fluorouracil (5-FU) had an average CC_50_ value of 31.0 µM and was thus much weaker than most of the compounds in series **1** and **2**. For example, **1k** had an average potency figure that was 310 times lower than the figure for 5-FU. Doxorubicin is an established potent anticancer drug with an average CC_50_ figure of 0.25 µM, which is a higher figure than was recorded for **1c**–**e**,**g**,**k**. In summary, the compounds in series **1** are potent cytotoxins.

A major issue in examining the potential of candidate anticancer agents is whether tumor-selective toxicity is displayed. In order to address this issue, the compounds in series **1** and **2** were evaluated against HGF, HPLF, and HPC non-malignant oral cells. The data generated are presented in Table 2. In general, the compounds had CC_50_ values in the low micromolar range. An exception was **1l,** which had very low toxicity to the non-malignant cells. This observation and the biodata for **1l** in Table 1 may be due to the strong electronegative properties of the 4-dimethylamino group, which has a Hammet sigma (σ) value of −0.83 [22]. This substituent will increase the electron density of the olefinic methine group, thereby reducing the electrophilicity of α,β-unsaturated ketones for cellular thiols.

Selectivity was ascertained as follows. Under clinical conditions, tumors were surrounded by a variety of non-malignant cells. Hence, selectivity was determined by dividing the average CC_50_ figure of the compound towards HGF, HPLF, and HPC cells by the CC_50_ value of a compound towards a specific neoplasm, which led to Selectivity Index (SI) figures. The SI values are presented in Table 1 and are all greater than 1, which indicated that in this case, the compounds displayed tumor-selective toxicity. The compounds with SI values over 50 towards a specific cell line were **1h** (HSC-2), **1k** (Ca9-22), **2a** (Ca9-22), and 5-FU (HSC-4). The compounds with the highest average SI values were **1g** (23.9), **1h** (22.0), **1k** (28.1), and **2a** (30.3). In summary, the compounds **1d**,**e**,**g**,**k** displayed excellent cytotoxicity to Ca9-22, HSC-2, HSC-3, and HSC-4 cells while **1g**,**h**,**k**, **2a** had noteworthy SI values.

The data presented so far indicate that the compounds in series **1** were potent cytotoxins towards a number of neoplasms. In addition, many of the compounds are far more toxic to neoplasms than to non-malignant cells. In order to identify lead compounds with both of these desirable attributes, potency–selectivity expression (PSE) values for each compound were generated and are listed in Table 2. The PSE values are the products of the reciprocal of the average CC_50_ value of the compound towards Ca9-22, HSC-2, HSC-3, and HSC-4 cells and the average SI value times 100. The amides **1d**,**g**,**k** had the highest PSE figures; in particular, **1k** had an outstanding PSE figure and was clearly a lead molecule. In regard to the compounds with no aryl substituents, namely **1a**, **2a**,**b**, *N*-acylation (as in **1a**, **2b**) led to compounds with lower PSE values than the parent compound **2a**. 

A study was undertaken to evaluate whether one or more physicochemical constants of the aryl substituents correlated with the cytotoxic potency and selective toxicity displayed by the compounds in series **1**. The physicochemical constants chosen were the Hammett sigma (σ) values, the Hansch pi (π) figures, and the molar refractivity (MR) values, which represented the electronic, hydrophobic, and steric properties, respectively, of the aryl substituents.

In order to probe for any correlations, the following sequence of graphs were constructed. The sigma, pi, and MR values were taken from the literature [22].

1. Linear graphs were made between the average CC_50_ values of **1a**–**l** and the σ values, the π constants, and subsequently with the MR figures.
2. Linear graphs were prepared between the average SI values of **1a**–**l** and the σ, π, and MR constants.
3. Linear graphs were made between the PSE values of **1a**–**l** and the σ, π, and MR constants.
4. Stages 1–3 were repeated, except semilogarithmic were made, not linear plots.
5. Stages 1–3 were repeated, except correlations were sought with the data for **1a**–**k**, i.e., the outlier **1l** was removed from consideration.

Correlations noted (*p* < 0.05) are recorded in Table 3.

The results in Table 3 reveal that as the magnitude of the σ values increased in **1a**–**l,** the potency rose. The PSE data (which takes into consideration the σ values) also increased with the more electron-attracting substituents. Removal of the outlier **1l** from consideration revealed that the potency of **1a**–**k** was related to the electronic properties of the aryl groups. In the future, groups with strongly electron attracting properties should be placed in the aryl rings, such as the 3,4-dinitro and 3-cyano-4-nitro groups, which had combined σ values of 1.49 and 1.34, respectively [18].

The next phase of the investigation involved attempts to find some of the ways whereby cytotoxicity occurs. The average CC_50_ values of **1a**–**l** towards Ca9-22, HSC-2, HSC-3, and HSC-4 cells were 0.21, 0.26, 0.58, and 0.48 µM, respectively. These results indicate the good sensitivity of Ca9-22 cells to this series of compounds and the mode of action studies used this cell line.

The first experiment was designed to assess whether representative compounds in series **1** interfered with the cell cycle. The three compounds with the lowest CC_50_ values towards Ca9-22 cells were **1d**,**e**,**k**. Four concentrations of each compound were used. The data generated are presented in Figure 3. Concentrations of 0.1, 0.2, and 0.4 µM of **1d**,**e**,**k** had little effects on the cell cycle, but at the highest concentration of 0.8 µM, a significant effect was noted (*p* < 0.05), and the most prominent increase of G2M cell population occurred with the three compounds (indicated by red arrows). Only a slight increase of the subG1 population occurred by **1e** and **1k** at 0.8 µM, but much lower than that was achieved by actinomycin D (1 µM) (Figure 3). 

Another way in which cytotoxicity could occur is by initiating mitosis. In order to examine this possibility, different concentrations of the potent cytotoxin **1k** that were 3, 10, and 30 times the CC_50_ value were incubated with Ca9-22 cells for 20 h. The result is portrayed in Figure 4, which revealed that at the two highest concentrations of **1k**, the percentage of the cells undergoing mitosis increased significantly. In order to explore the possibility that the dichloroacetyl group contributed to this effect, concentrations of **2a** that were 3, 10, and 30 times the CC_50_ concentration were incubated with Ca9-22 cells for 20 h. The data in Figure 4 reveals that mitotic accumulation occurred with 2 µM and 6 µM of **2a** were employed. The question arose as to the way in which this observation could be useful in anticancer drug design. Cells which are stimulated to divide are likely more sensitive to a subsequent attack of a cytotoxin when cells are in a resting stage, for example. In fact, the mitotic accumulation on Ca9-22 cells may be a contributor to the greater toxicity of series **1** and **2** to neoplasms than to non-malignant cells, e.g., **1k** had a SI value of 72.0 (Table 1).

Poly(ADP-ribose) polymerase (PARP) enzymes are known to be involved in the cellular response to DNA damage. PARP first detects DNA strand breaks and initiates the repair pathway through the modulation of chromatin structure and interaction with DNA repair factors [23]. Hence, inhibition of PARP may be useful in treating neoplastic conditions. The data in Figure 5 reveals that at concentrations of 0.2 and 0.6 µM of **1k** and **2** and 6 µM of **2a**, PARP was cleaved, which may have contributed to its cytotoxic effect. Furthermore, procaspase 3 may be cleaved to liberate caspase 3, and this result in turn led to apoptotic cell death. As may be seen from Figure 4, at concentrations of 0.2 and 0.6 µM of **1k** and **2** and 6 µM of **2a**, cleavage of procaspase to caspase 3 was observed. However, it should be noted that these compounds showed more prominent mitotic and G2/M accumulation of cell cycle, in contrast with actinomycin D, a positive control of apoptosis inducer.

## 5. Conclusions

**Figure 6:**
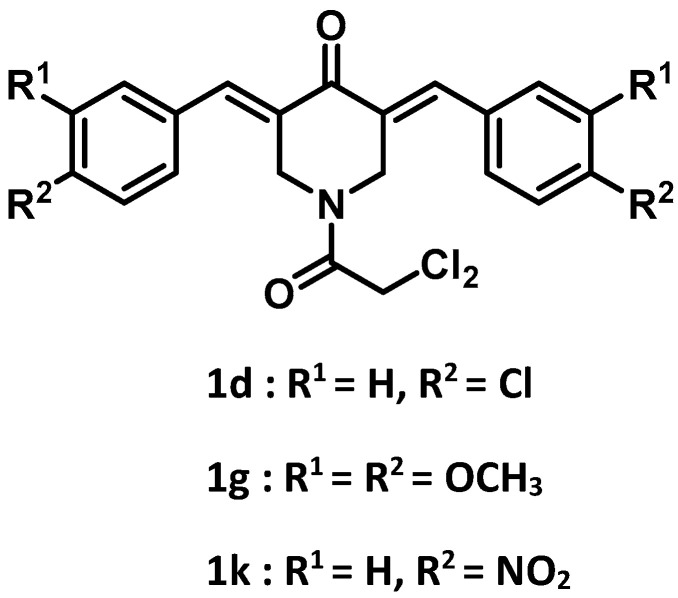
The structures of three lead molecules **1d** (R^1^ = H, R^2^ = Cl), **1g** (R^1^ = R^2^ = OCH_3_), and **1k** (R^1^ = H, R^2^ = NO_2_).

This study revealed that, in general, the compounds in series **1** displayed potent cytotoxicity towards a number of squamous cell carcinomas, inducing prominent mitotic or G2/M accumulation rather than apoptosis. These compounds were less toxic to some non-malignant cells. From this study, **1d**,**g** and especially **1k** emerged as lead molecules for future development. The structures of these compounds are presented in Figure 6. In addition to the design of analogs with highly electron-attracting aryl substituents *vide supra*, the evaluation of bioisosteric analogs, such as the 4-bromo and 2,6-dichloro compounds, should be considered. In the future, efforts should be made to find additional ways in which cytotoxicity is displayed, such as whether the compounds interfere with proteasomes in malignant cells.

## Figures and Tables

**Table 1:** Evaluation of **1a**–**l, 2a**,**b** against Ca9-22, HSC-2, HSC-3, and HSC-4 cancer cells.

Compound	Aryl Group	Ca9-22 CC_50_ (µM)	SI ^a^	HSC-2 CC_50_ (µM)	SI ^a^	HSC-3 CC_50_ (µM)	SI ^a^	HSC-4 CC_50_ (µM)	SI ^a^	Average CC_50_ (µM)	SI ^a^
**1a**	H	0.42 ± 0.04	9.64	0.67 ± 0.06	6.05	1.29 ± 0.07	3.14	0.59 ± 0.12	6.86	0.74	6.42
**1b**	4-F	0.11 ± 0.01	19.8	0.25 ± 0.02	8.72	0.44 ± 0.02	4.96	0.33 ± 0.07	6.61	0.28	10.0
**1c**	3,4-F_2_	0.09 ± 0.02	20.0	0.20 ± 0.02	9.00	0.35 ± 0.02	5.14	0.20 ± 0.01	9.00	0.21	10.8
**1d**	4-Cl	0.05 ± 0.00	28.8	0.10 ± 0.01	14.4	0.19 ± 0.02	7.58	0.18 ± 0.04	8.00	0.13	14.7
**1e**	3,4-Cl_2_	0.04 ± 0.00	19.5	0.08 ± 0.01	9.75	0.13 ± 0.06	6.00	0.23 ± 0.01	3.39	0.12	9.66
**1f**	4-OCH_3_	0.39 ± 0.02	15.4	0.52 ± 0.07	11.5	1.00 ± 0.16	5.99	1.26 ± 0.27	4.75	0.79	9.41
**1g**	3,4-(OCH_3_)_2_	0.11 ± 0.02	36.8	0.17 ± 0.02	23.8	0.28 ± 0.03	14.5	0.20 ± 0.04	20.3	0.19	23.9
**1h**	3,4,5-(OCH_3_)_3_	0.72 ± 0.15	11.9	0.14 ± 0.01	61.3	1.37 ± 0.22	6.26	0.99 ± 0.39	8.67	0.80	22.0
**1i**	3,4-OCH_2_O	0.17 ± 0.06	25.1	0.36 ± 0.01	11.9	0.74 ± 0.13	5.77	0.65 ± 0.17	6.57	0.48	12.3
**1j**	4-CH_3_	0.15 ± 0.03	32.1	0.26 ± 0.01	18.5	0.46 ± 0.04	10.5	0.45 ± 0.04	10.7	0.33	18.0
**1k**	4-NO_2_	0.02 ± 0.01	72.0	0.08 ± 0.01	18.0	0.10 ± 0.03	14.4	0.18 ± 0.04	8.00	0.10	28.1
**1l**	4-N(CH_3_)_2_	13.6 ± 2.70	10.3	14.9 ± 4.40	9.40	45.5 ± 7.60	3.08	19.6 ± 6.30	7.14	23.4	7.48
**2a**	-	0.19 ± 0.03	61.6	0.39 ± 0.01	30.0	0.80 ± 0.07	14.6	0.79 ± 0.22	14.8	0.54	30.3
**2b**	-	0.64 ± 0.01	17.3	1.23 ± 0.07	9.02	2.25 ± 0.08	4.93	1.57 ± 0.12	7.07	1.42	9.58
SDA ^b^		>200	-	>200	-	>200	-	>200	-	>200	-
5-FU ^c^		24.5 ± 12.3	>40.7	30.5 ± 7.1	>32.7	61.3 ± 9.8	>16.3	7.58 ± 0.5	>131	31.0	>55.2
DXR ^d^		0.43 ± 0.04	>22.2	0.20 ± 0.02	>47.8	0.26 ± 0.21	>36.7	0.12 ± 0.00	>79.6	0.25	>46.6

^a^ The letters SI refer to the selectivity index. These figures are generated by dividing the average CC_50_ value of the compound towards HGF, HPLF, and HPC (Table 2) by the CC_50_ figure of the compound against a specific neoplastic cell line. Each CC_50_ value represents mean ± S.D. of triplicate determinations. ^b^ The letters SDA refer to sodium dichloroacetate. ^c^ 5-FU refers to 5-fluorouracil. ^d^ DXR means doxorubicin.

**Table 2:** Evaluation of **1a**–**l** and **2a**,**b** against human HGF, HPLF, and HPC non-malignant cells.

Compound	Aryl Group	CC_50_ (µM)				PSE ^a^
		HGF	HPLF	HPC	Average	
**1a**	H	5.90 ± 0.10	3.37 ± 0.42	2.88 ± 0.08	4.05	868
**1b**	4-F	2.97 ± 0.46	2.26 ± 0.11	1.30 ± 0.16	2.18	3571
**1c**	3,4-F_2_	2.21 ± 0.11	1.91 ± 0.08	1.28 ± 0.37	1.80	5143
**1d**	4-Cl	2.13 ± 0.12	1.29 ± 0.14	0.90 ± 0.02	1.44	11,308
**1e**	3,4-Cl_2_	1.04 ± 0.13	0.82 ± 0.10	0.49 ± 0.00	0.78	8050
**1f**	4-OCH_3_	9.03 ± 0.76	5.33 ± 0.32	3.62 ± 0.07	5.99	1191
**1g**	3,4-(OCH_3_)_2_	8.13 ± 2.47	2.83 ± 1.53	1.19 ± 0.09	4.05	12,579
**1h**	3,4,5-(OCH_3_)_3_	4.70 ± 1.60	17.0 ± 5.10	4.00 ± 0.60	8.58	2750
**1i**	3,4-OCH_2_O	5.67 ± 0.31	4.37 ± 0.85	2.78 ± 0.14	4.27	2563
**1j**	4-CH_3_	5.53 ± 0.31	5.00 ± 0.10	3.93 ± 0.65	4.82	5455
**1k**	4-NO_2_	3.01 ± 0.33	0.76 ± 0.13	0.56 ± 0.08	1.44	28,100
**1l**	4-N(CH_3_)_2_	111 ± 27.0	188 ± 21.0	122 ± 69.0	140	32.0
**2a**	H	21.7 ± 6.00	9.10 ± 2.87	4.30 ± 0.72	11.7	5611
**2b**	H	23.0 ± 1.70	5.60 ± 1.13	4.83 ± 0.12	11.1	675
SDA ^b^		>200	>200	>200	>200	-
5-FU ^c^		>1000	>1000	>987	>996	>178
DXR ^d^		>10	>10	>8.65	>9.55	>18,640

^a^ The letters PSE refer to the potency–selectivity expression. These values are the products of the reciprocal of the average CC_50_ values of the compounds towards Ca9-22, HSC-2, HSC-3, and HSC-4 cells and the average SI values × 100. Each CC_50_ value represents mean ± S.D. of triplicate assays. ^b^ The letters SDA refer to sodium dichloroacetate. ^c^ 5 -FU means 5-flurouracil. ^d^ The letters DXR refer to doxorubicin.

**Table 3:** Correlations noted (*p* < 0.05) when the σ, π, and MR constants of the aryl substituents were plotted against the average CC_50_ values, average SI figures, and PSE values.

Plot	Compounds	Correlations
Linear	**1a–l**	σ (−ve), PSE (+ve)
Semilogarithmic	**1a–l**	σ (−ve), PSE (+ve)
Linear	**1a–k**	PSE (+ve)
Semilogarithmic	**1a–k**	σ (−ve), PSE (+ve)

## Data Availability

The ^1^H, ^13^C NMR spectra and mass spectra of compounds in series **1** and **2** are available in the Supplementary section of this article.

## Supplementary Materials

The following supporting information can be downloaded at: https://www.mdpi.com/article/10.3390/medicines9060035/s1.
