# Supplementary material for: Dichloroacetyl Amides of 3,5-Bis(benzylidene)-4-piperidones Displaying Greater Toxicity to Neoplasms than to Non-Malignant Cells
Source: Medicines (Basel). 2022 Jun 8;9(6):35. doi: 10.3390/medicines9060035 (PMC9228592; doi:10.3390/medicines9060035)
Supplement: Supplementary file 1 [file medicines-09-00035-s001.zip › medicines-1705488-supplementary.pdf]

SUPPLEMENTAL SECTION

Dichloroacetyl amides of 3,5-bis(benzylidene)-4-piperidones displaying greater toxicity to neoplasms than non-malignant cells

Mohammad Hossain [a], Praveen K. Roayapalley [b,*], Hiroshi Sakagami [c], Keitaro Satoh [c], Kenjiro Bandow [c], Umashankar Das [b], Jonathan R. Dimmock [b]

[a] *School of Sciences, Indiana University Kokomo, Kokomo, IN 46904, USA*

[b] *Drug Discovery and Development Research Cluster, University of Saskatchewan, Saskatoon, Saskatchewan S7N 5E5, Canada*

[c] *Meikai University Research Institute of Odontology, Sakado, Saitama 350-0283, Japan*

*Corresponding author: E-mail : rpraveen.sp@usask.ca Tel.: +1-306-715-4217

Contents

^{1}H NMR (($CDCl_3$) spectrum of (3*E*,5*E*)-3,5-dibenzylidene-1-(2,2-dichloroacetyl)piperidin-4-one (**1a**) 4

^{13}C NMR ($CDCl_3$) spectrum of (3E,5E)-3,5-dibenzylidene-1-(2,2-dichloroacetyl)piperidin-4-one (**1a**) 5

Mass spectrum of (3*E*,5*E*)-3,5-dibenzylidene-1-(2,2-dichloroacetyl)piperidin-4-one (**1a**) 6

^{1}H NMR ($CDCl_3$) spectrum of 1-(2,2-dichloroacetyl)-3,5-bis((*E*)-4-fluorobenzylidene)piperidin-4-one (**1b**) 7

^{13}C NMR ($CDCl_3$) spectrum of 1-(2,2-dichloroacetyl)-3,5-bis((*E*)-4-fluorobenzylidene)piperidin-4-one (**1b**) 8

Mass spectrum of 1-(2,2-dichloroacetyl)-3,5-bis((*E*)-4-fluorobenzylidene)piperidin-4-one (**1b**) 9

^{1}H NMR ($CDCl_3$) spectrum of 1-(2,2-dichloroacetyl)-3,5-bis((*E*)-3,4-difluorobenzylidene)piperidin-4-one (**1c**) 10

^{13}C NMR ($CDCl_3$) spectrum of 1-(2,2-dichloroacetyl)-3,5-bis((*E*)-3,4-difluorobenzylidene)piperidin-4-one (**1c**) 11

Mass spectrum of 1-(2,2-dichloroacetyl)-3,5-bis((*E*)-3,4-difluorobenzylidene)piperidin-4-one (**1c**) 12

^{1}H NMR ($CDCl_3$) spectrum of 3,5-bis((*E*)-4-chlorobenzylidene)-1-(2,2-dichloroacetyl)piperidin-4-one (**1d**) 13

^{13}C NMR ($CDCl_3$) spectrum of 3,5-bis((*E*)-4-chlorobenzylidene)-1-(2,2-dichloroacetyl)piperidin-4-one (**1d**) 14

Mass spectrum of 3,5-bis((*E*)-4-chlorobenzylidene)-1-(2,2-dichloroacetyl)piperidin-4-one (**1d**) 15

^{1}H NMR ($CDCl_3$) spectrum of 1-(2,2-dichloroacetyl)-3,5-bis((*E*)-3,4-dichlorobenzylidene)piperidin-4-one (**1e**) 16

^{13}C NMR ($CDCl_3$) spectrum of 1-(2,2-dichloroacetyl)-3,5-bis((*E*)-3,4-dichlorobenzylidene)piperidin-4-one (**1e**) 17

Mass spectrum of 1-(2,2-dichloroacetyl)-3,5-bis((*E*)-3,4-dichlorobenzylidene)piperidin-4-one (**1e**) 18

^{1}H NMR ($CDCl_3$) spectrum of 1-(2,2-dichloroacetyl)-3,5-bis((*E*)-4-methoxybenzylidene)piperidin-4-one (**1f**) 19

^{13}C NMR ($CDCl_3$) spectrum of 1-(2,2-dichloroacetyl)-3,5-bis((*E*)-4-methoxybenzylidene)piperidin-4-one (**1f**) 20

Mass spectrum of 1-(2,2-dichloroacetyl)-3,5-bis((*E*)-4-methoxybenzylidene)piperidin-4-one (**1f**) 21

^{1}H NMR ($(CD_3)_2SO$) spectrum of 1-(2,2-dichloroacetyl)-3,5-bis((*E*)-3,4-dimethoxybenzylidene)piperidin-4-one (**1g**) 22

^{13}C NMR ($(CD_3)_2SO$) spectrum of 1-(2,2-dichloroacetyl)-3,5-bis((*E*)-3,4-dimethoxybenzylidene)piperidin-4-one (**1g**) 23

Mass spectrum of 1-(2,2-dichloroacetyl)-3,5-bis((*E*)-3,4-dimethoxybenzylidene)piperidin-4-one (**1g**) 24

^{1}H NMR ($(CD_3)_2SO$) spectrum of 1-(2,2-dichloroacetyl)-3,5-bis((*E*)-3,4,5-trimethoxybenzylidene)piperidin-4-one (**1h**) 25

^{13}C NMR ($(CD_3)_2SO$) spectrum of 1-(2,2-dichloroacetyl)-3,5-bis((*E*)-3,4,5-trimethoxybenzylidene)piperidin-4-one (**1h**) 26

Mass spectrum of 1-(2,2-dichloroacetyl)-3,5-bis((*E*)-3,4,5-trimethoxybenzylidene)piperidin-4-one (**1h**) 27

^{1}H NMR ($(CD_3)_2SO$) spectrum of 1-(2,2-dichloroacetyl)-3,5-bis((*E*)-3,4-methylenedioxybenzylidene)piperidin-4-one (**1i**) 28
^{13}C NMR ($(CD_3)_2SO$) spectrum of 1-(2,2-dichloroacetyl)-3,5-bis((*E*)-3,4-methylenedioxybenzylidene)piperidin-4-one (**1i**) 29
Mass spectrum of 1-(2,2-dichloroacetyl)-3,5-bis((*E*)-3,4-methylenedioxybenzylidene)piperidin-4-one (**1i**)... 30
^{1}H NMR ($CDCl_3$) spectrum of 1-(2,2-dichloroacetyl)-3,5-bis((*E*)-4-methylbenzylidene)piperidin-4-one (**1j**)... 31
^{13}C NMR ($CDCl_3$) spectrum of 1-(2,2-dichloroacetyl)-3,5-bis((*E*)-4-methylbenzylidene)piperidin-4-one (**1j**).. 32
Mass spectrum of 1-(2,2-dichloroacetyl)-3,5-bis((*E*)-4-methylbenzylidene)piperidin-4-one (**1j**)... 33
^{1}H NMR ($(CD_3)_2SO$) spectrum of 1-(2,2-dichloroacetyl)-3,5-bis((*E*)-4-nitrobenzylidene)piperidin-4-one (**1k**) 34
^{13}C NMR ($(CD_3)_2SO$) spectrum of 1-(2,2-dichloroacetyl)-3,5-bis((*E*)-4-nitrobenzylidene)piperidin-4-one (**1k**) 35
Mass spectrum of 1-(2,2-dichloroacetyl)-3,5-bis((*E*)-4-nitrobenzylidene)piperidin-4-one (**1k**)... 36
^{1}H NMR ($CDCl_3$) spectrum of 1-(2,2-dichloroacetyl)-3,5-bis((*E*)-4-(dimethylamino)benzylidene)piperidin-4-one (**1l**) 37
^{13}C NMR ($CDCl_3$) spectrum of 1-(2,2-dichloroacetyl)-3,5-bis((*E*)-4-(dimethylamino)benzylidene)piperidin-4-one (**1l**) 38
Mass spectrum of 1-(2,2-dichloroacetyl)-3,5-bis((E)-4-(dimethylamino)benzylidene)piperidin-4-one (**1l**) 39
^{1}H NMR ($(CD_3)_2SO$) spectrum of 3,5-di((E)-benzylidene)piperidin-4-one (**2a**) 40
^{13}C NMR ($(CD_3)_2SO$) spectrum of 3,5-di((E)-benzylidene)piperidin-4-one (**2a**) 41
Mass spectrum of 3,5-di((E)-benzylidene)piperidin-4-one (**2a**).. 42
^{1}H NMR ($(CD_3)_2SO$) of 1-acetyl-3,5-di((E)-benzylidene)piperidin-4-one (**2b**).. 43
^{13}C NMR ($(CD_3)_2SO$) spectrum of 1-acetyl-3,5-di((E)-benzylidene)piperidin-4-one (**2b**)... 44
Mass spectrum of 1-acetyl-3,5-di((E)-benzylidene)piperidin-4-one (**2b**) 45

^{1}H NMR ($CDCl_3$) spectrum of (3*E*,5*E*)-3,5-dibenzylidene-1-(2,2-dichloroacetyl)piperidin-4-one (**1a**)

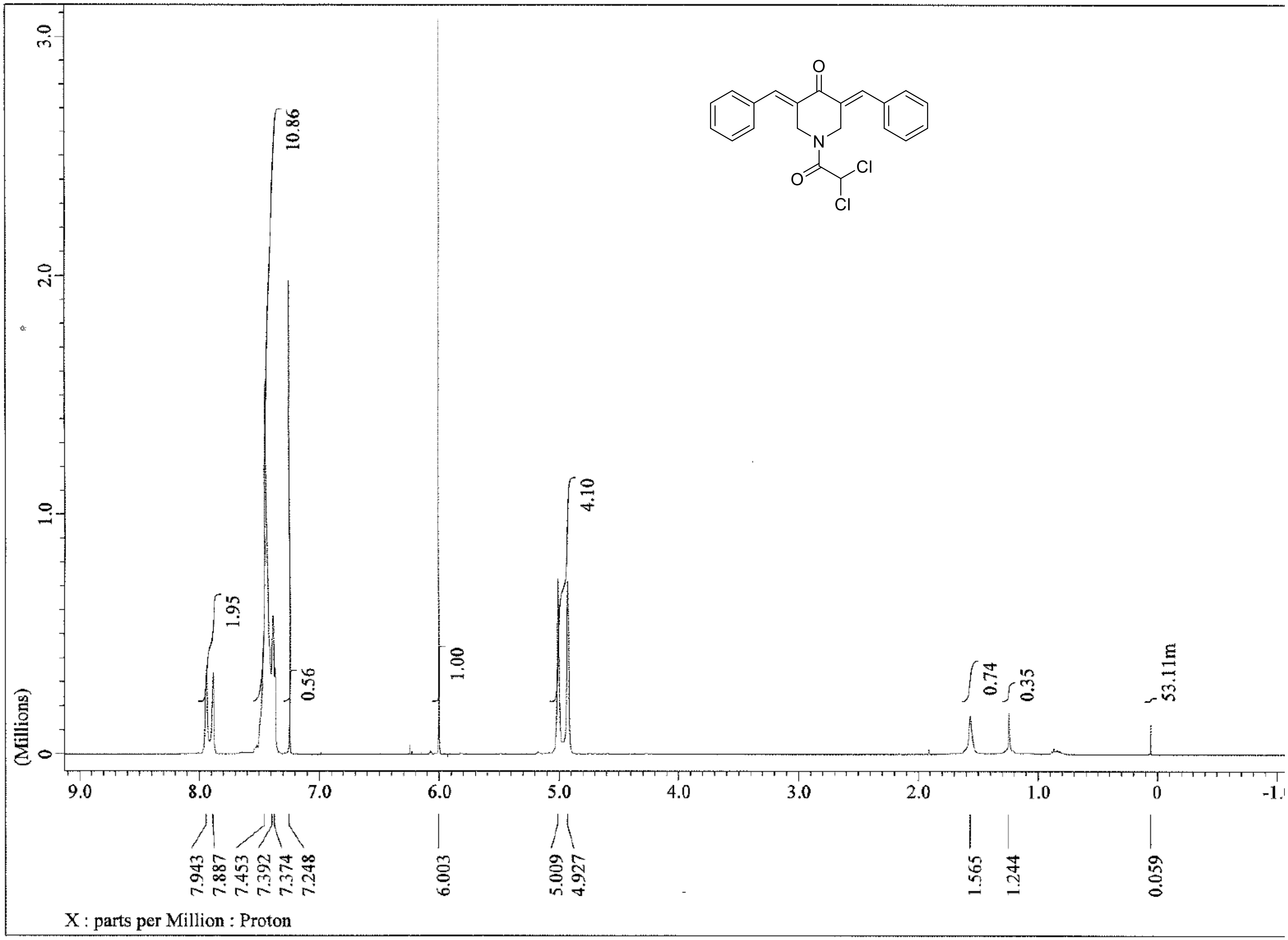

^{13}C NMR ($CDCl_3$) spectrum of (3E,5E)-3,5-dibenzylidene-1-(2,2-dichloroacetyl)piperidin-4-one (**1a**)

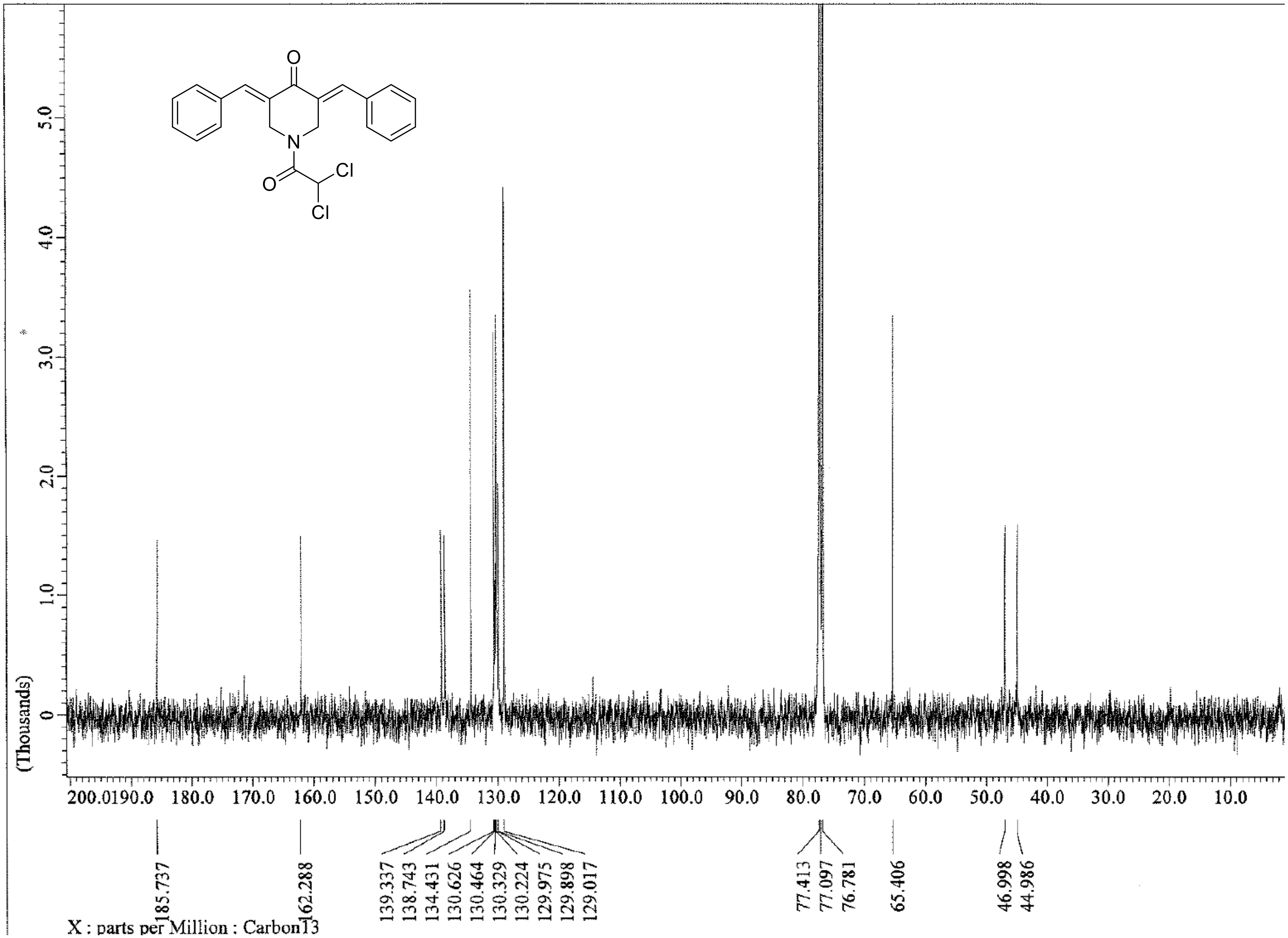

Mass spectrum of (3*E*,5*E*)-3,5-dibenzylidene-1-(2,2-dichloroacetyl)piperidin-4-one (**1a**)

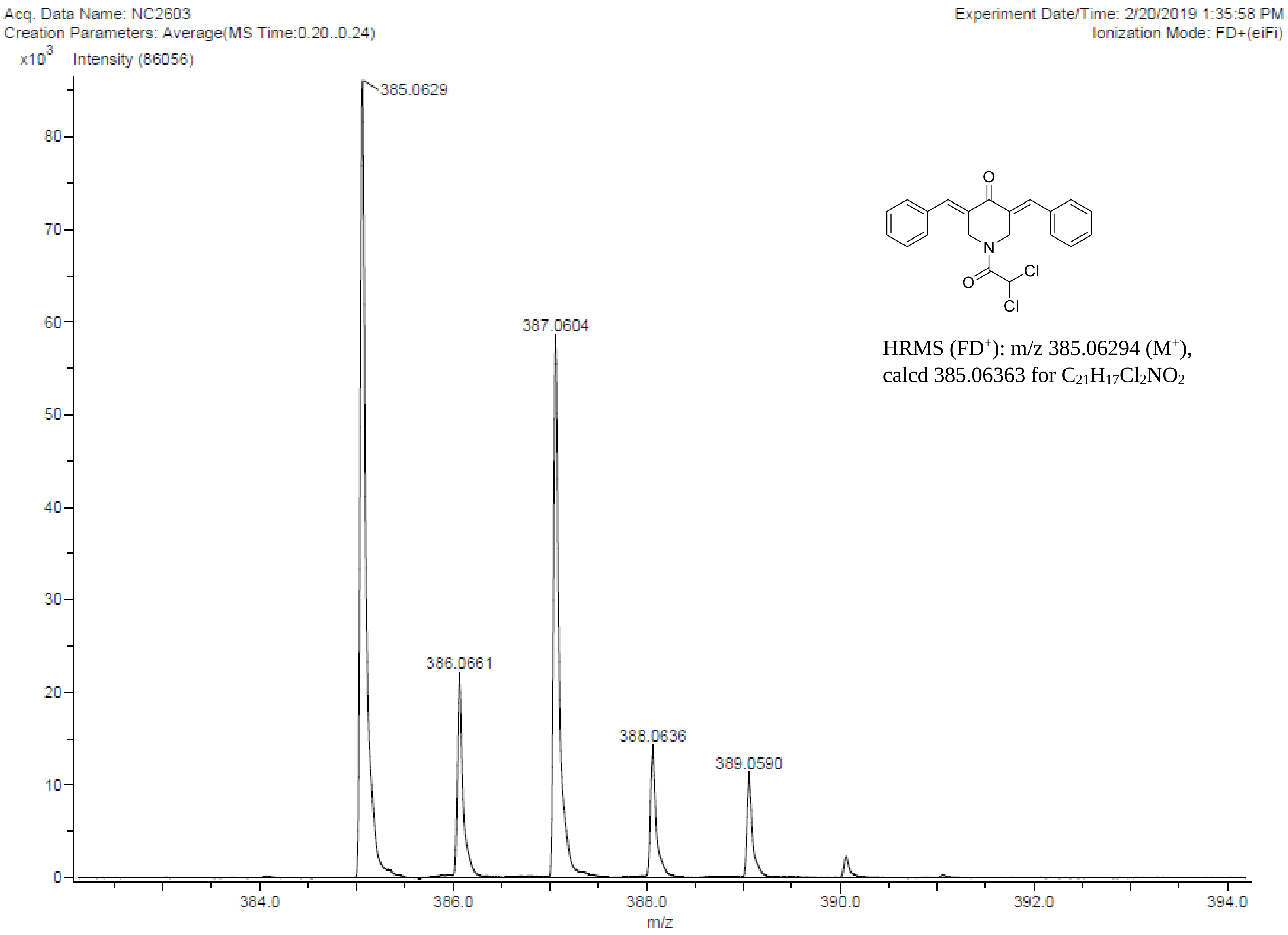

HRMS (FD^+): m/z 385.06294 (M^+), calcd 385.06363 for $C_{21}H_{17}Cl_2NO_2$

^{1}H NMR (CDCl$_3$) spectrum of 1-(2,2-dichloroacetyl)-3,5-bis((*E*)-4-fluorobenzylidene)piperidin-4-one (**1b**)

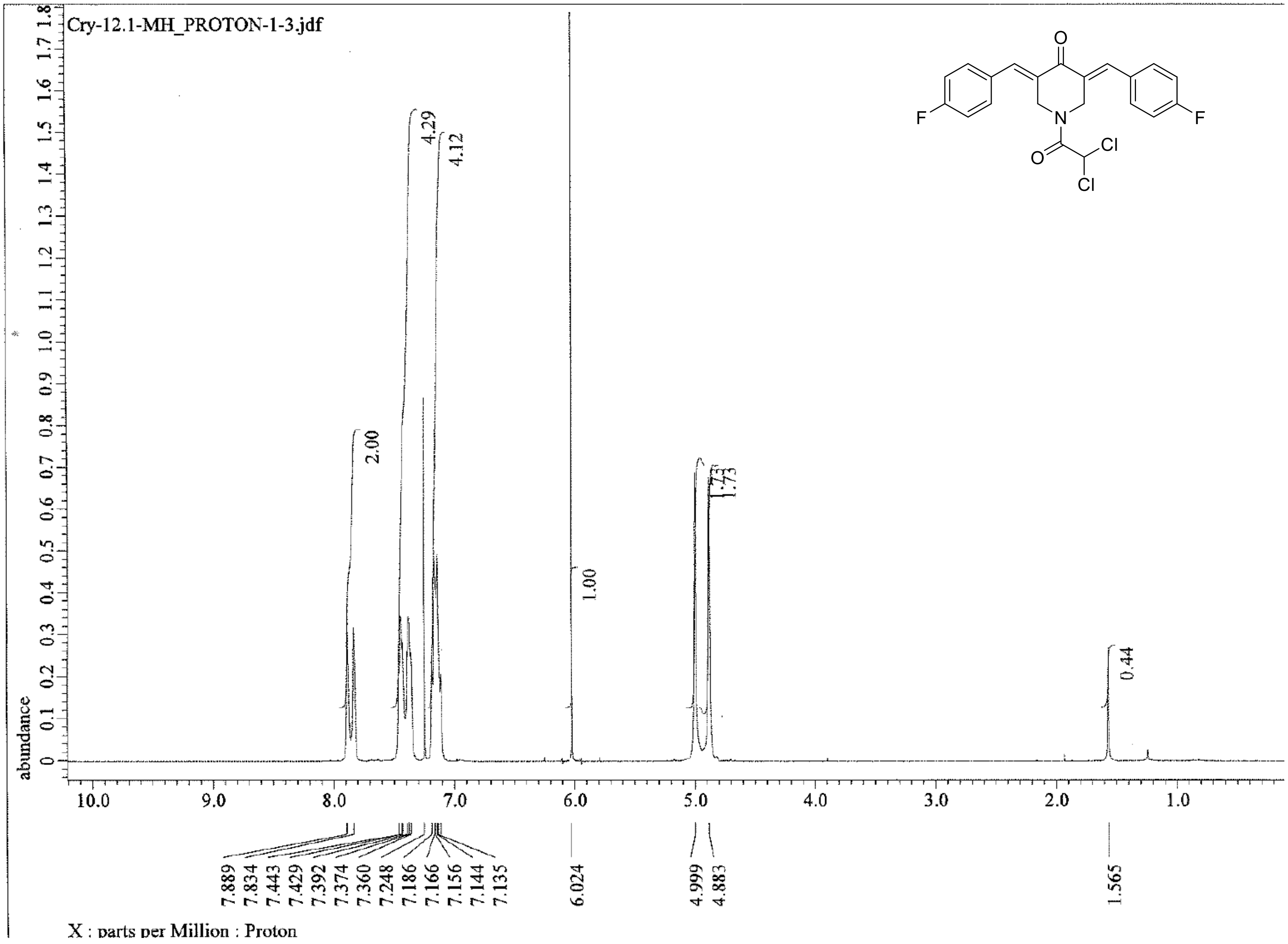

^{13}C NMR ($CDCl_3$) spectrum of 1-(2,2-dichloroacetyl)-3,5-bis((*E*)-4-fluorobenzylidene)piperidin-4-one (**1b**)

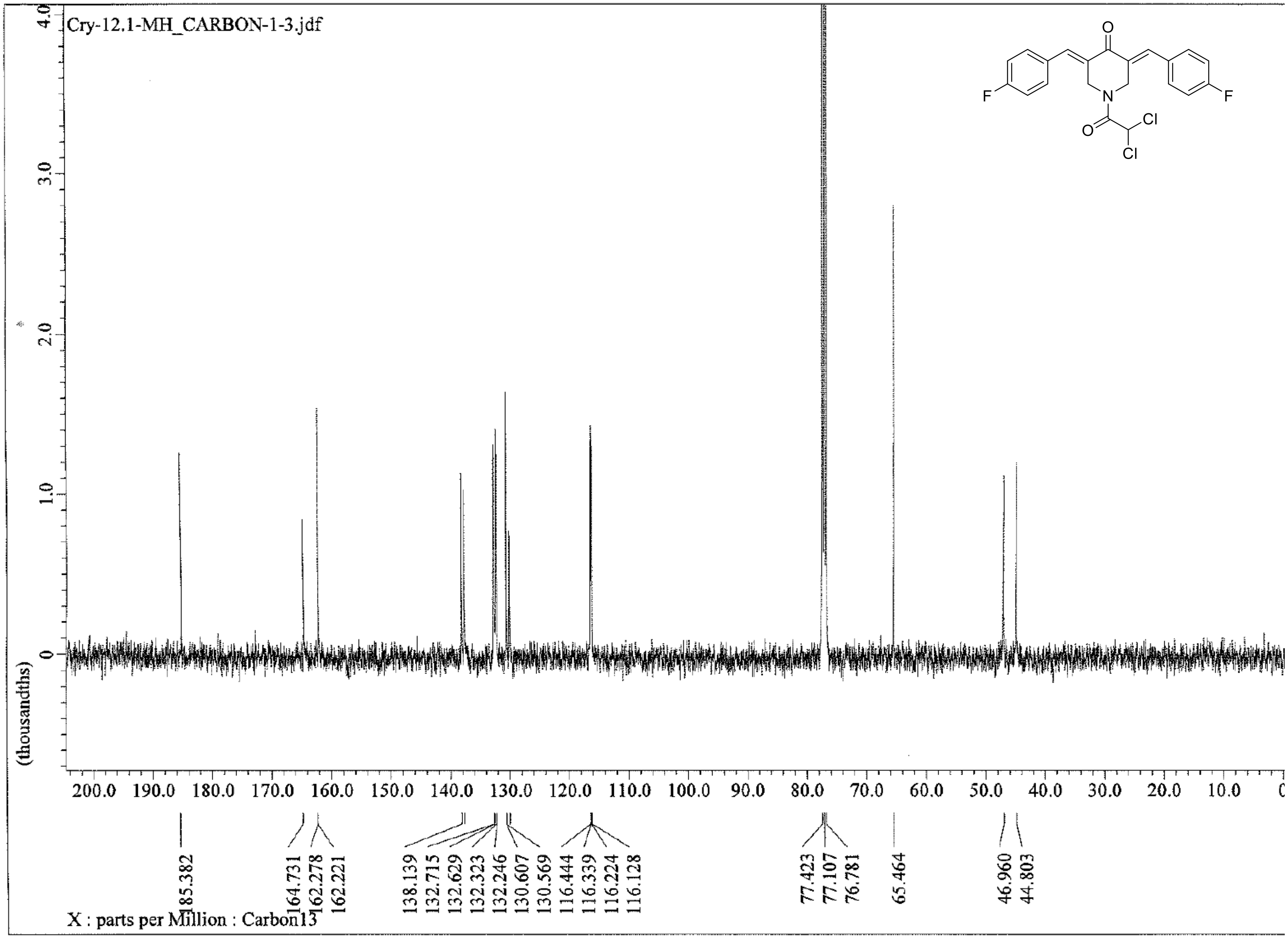

Mass spectrum of 1-(2,2-dichloroacetyl)-3,5-bis((*E*)-4-fluorobenzylidene)piperidin-4-one (**1b**)

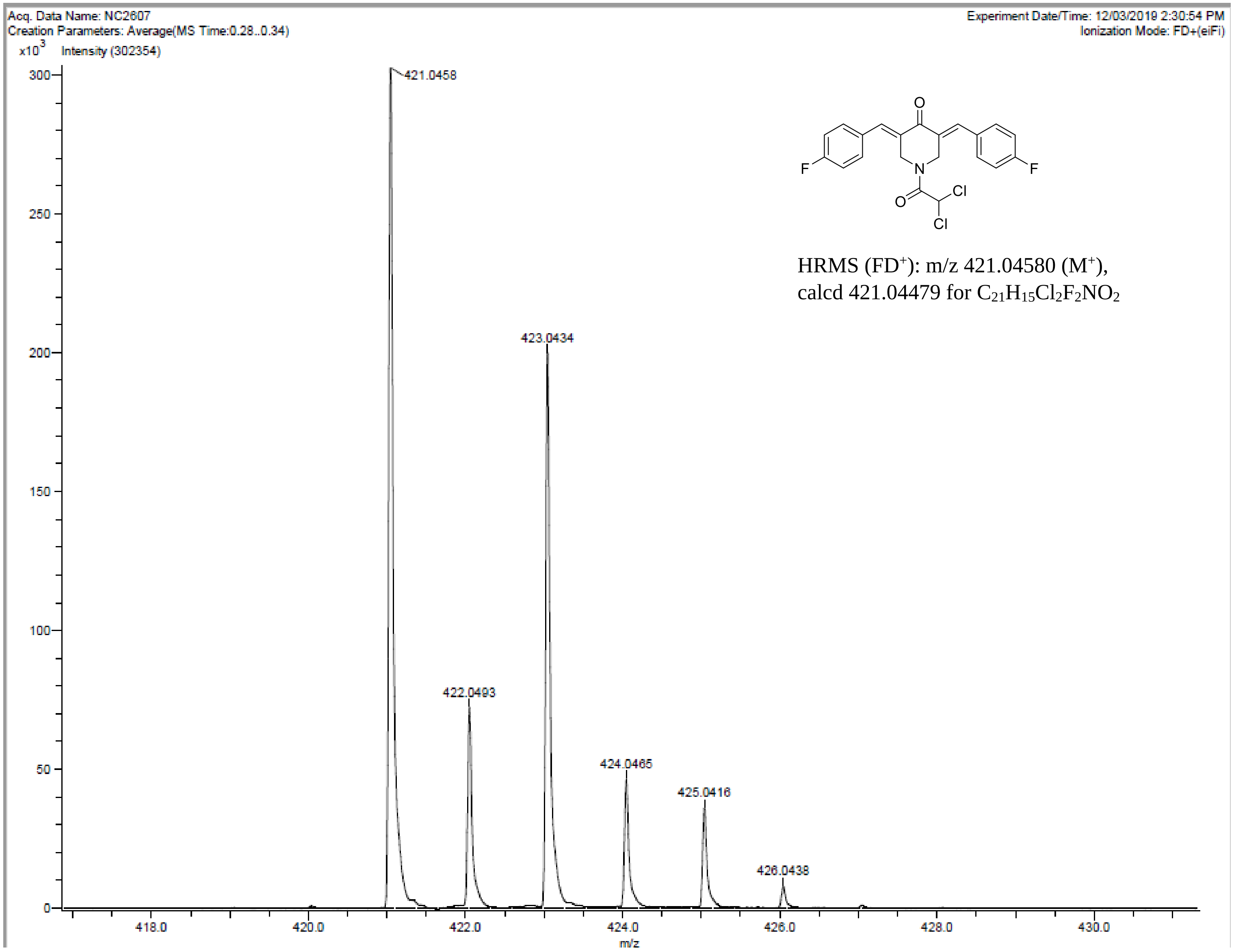

^{1}H NMR ($CDCl_3$) spectrum of 1-(2,2-dichloroacetyl)-3,5-bis((*E*)-3,4-difluorobenzylidene)piperidin-4-one (**1c**)

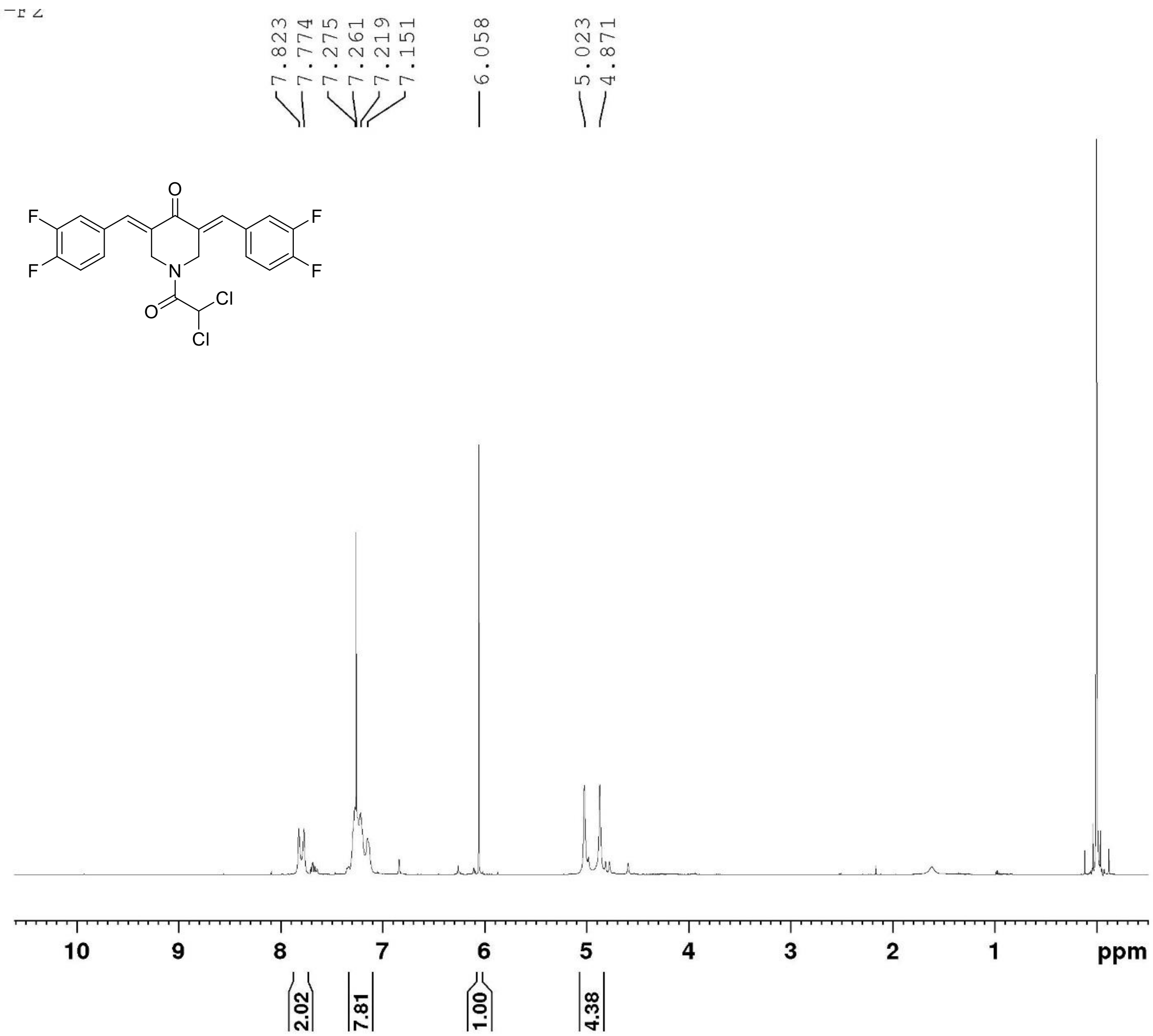

Current Data Parameters
NAME 08-23-2018
EXPNO 15
PROCNO 1

F2 - Acquisition Parameters
Date_ 20180824
Time 8.27
INSTRUM spect
PROBHD 5 mm PATXI 1H/
PULPROG zg30
TD 65536
SOLVENT CDCl3
NS 32
DS 2
SWH 10000.000 Hz
FIDRES 0.152588 Hz
AQ 3.2767999 sec
RG 90.5
DW 50.000 usec
DE 6.50 usec
TE 302.7 K
D1 1.00000000 sec
TD0 1

======== CHANNEL f1 ========
SFO1 500.1330885 MHz
NUC1 1H
P1 8.00 usec
PLW1 10.00000000 W

F2 - Processing parameters
SI 32768
SF 500.1300112 MHz
WDW EM
SSB 0
LB 0.30 Hz
GB 0
PC 1.00

^{13}C NMR ($CDCl_3$) spectrum of 1-(2,2-dichloroacetyl)-3,5-bis((*E*)-3,4-difluorobenzylidene)piperidin-4-one (**1c**)

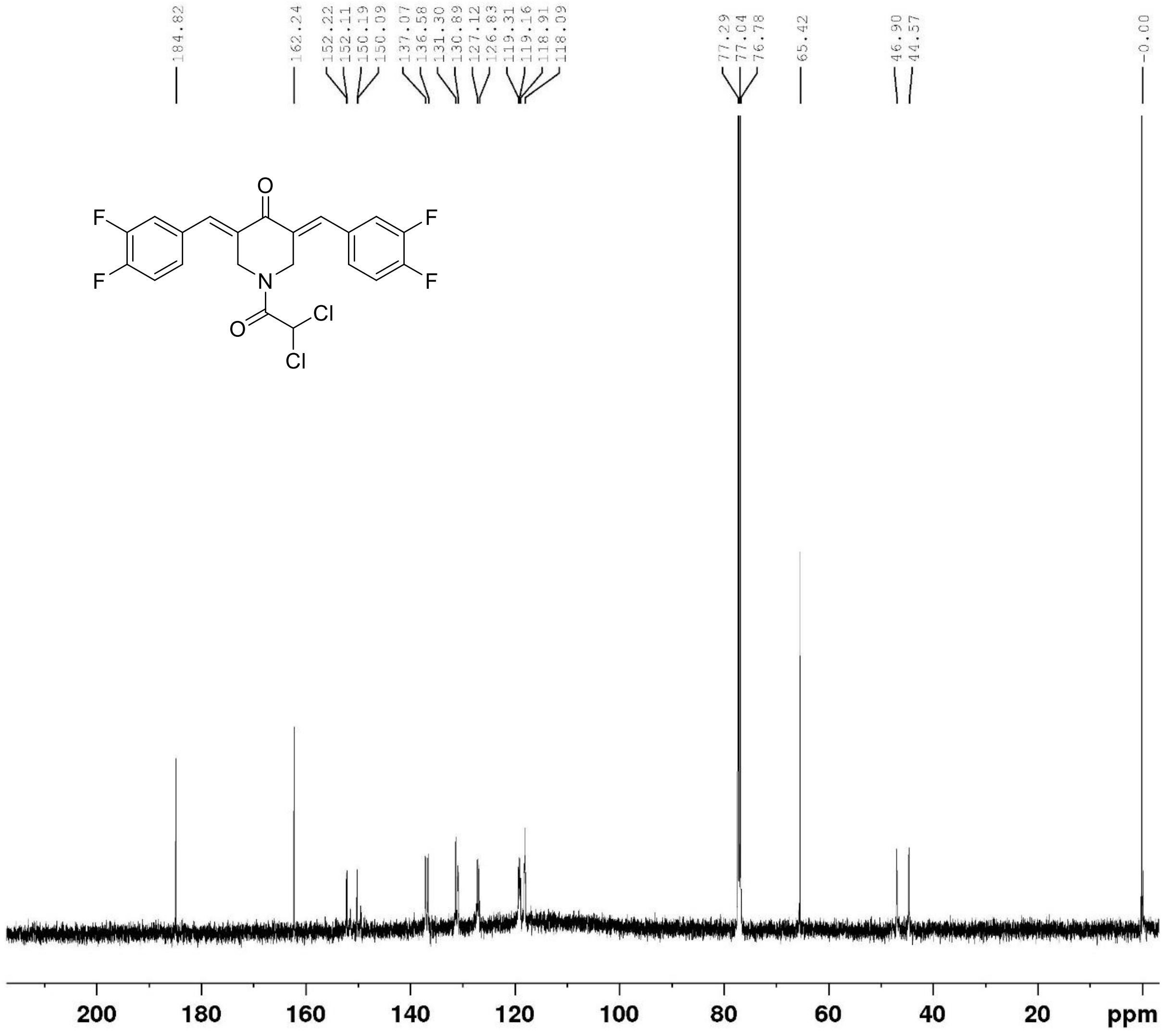

BRUKER

```
Current Data Parameters
NAME           08-23-2018
EXPNO                  16
PROCNO                  1

F2 - Acquisition Parameters
Date_            20180824
Time                10.16
INSTRUM             spect
PROBHD   5 mm PATXI 1H/
PULPROG           zgpg30
TD                  65536
SOLVENT             CDCl3
NS                   2048
DS                      4
SWH             29761.904 Hz
FIDRES           0.454131 Hz
AQ              1.1010048 sec
RG                    575
DW                 16.800 usec
DE                   6.50 usec
TE                  302.8 K
D1             2.00000000 sec
D11            0.03000000 sec
TD0                     1

======== CHANNEL f1 ========
SFO1          125.7703637 MHz
NUC1                  13C
P1                  12.00 usec
PLW1         180.00000000 W

======== CHANNEL f2 ========
SFO2          500.1320005 MHz
NUC2                   1H
CPDPRG[2          waltz16
PCPD2               80.00 usec
PLW2          10.00000000 W
PLW12          0.10000000 W
PLW13          0.06400000 W

F2 - Processing parameters
SI                  32768
SF            125.7577852 MHz
WDW                    EM
SSB       0
LB                   1.00 Hz
GB        0
PC                   1.40
```

Mass spectrum of 1-(2,2-dichloroacetyl)-3,5-bis((*E*)-3,4-difluorobenzylidene)piperidin-4-one (**1c**)

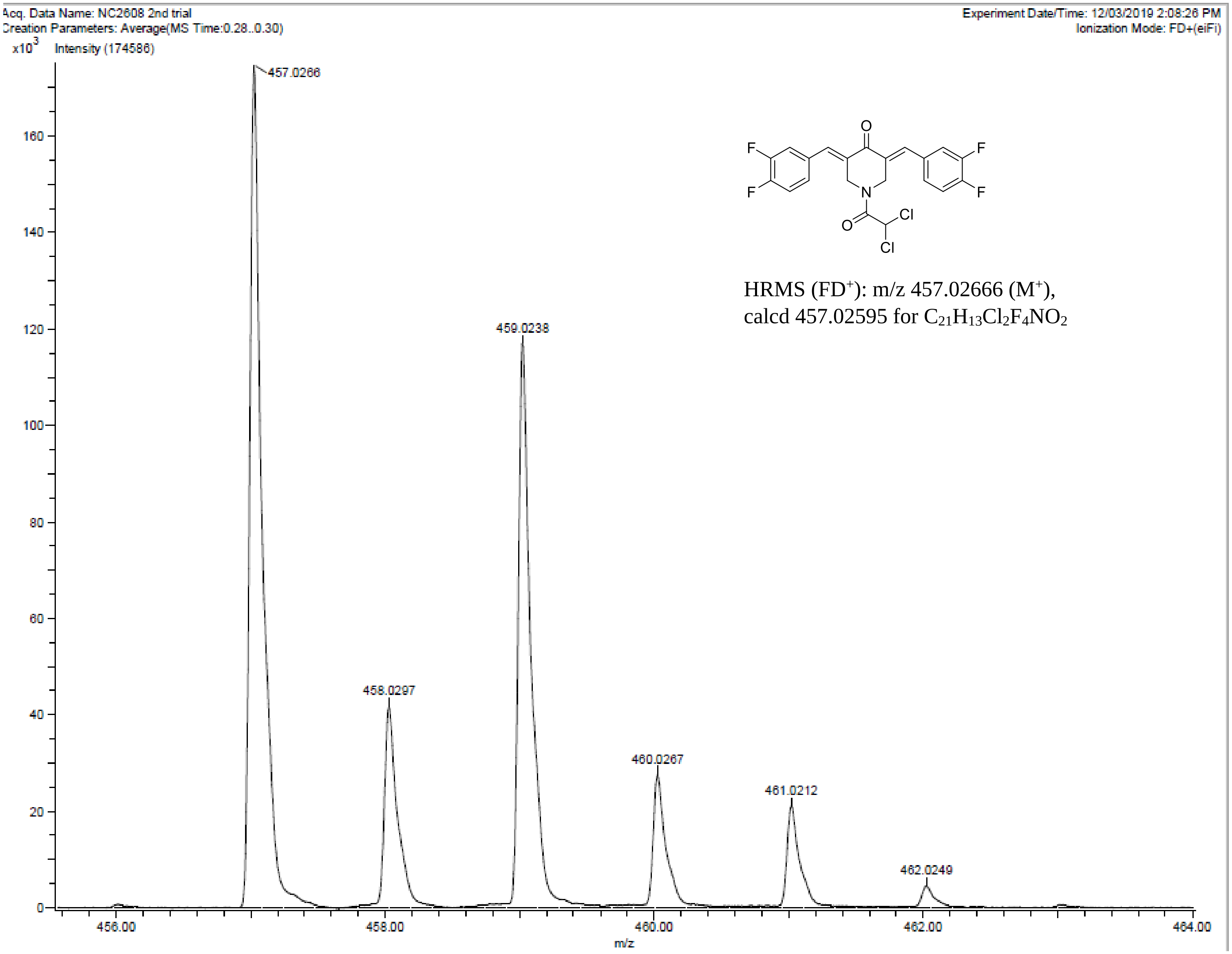

HRMS (FD$^+$): m/z 457.02666 (M$^+$), calcd 457.02595 for $C_{21}H_{13}Cl_2F_4NO_2$

^{1}H NMR ($CDCl_3$) spectrum of 3,5-bis((*E*)-4-chlorobenzylidene)-1-(2,2-dichloroacetyl)piperidin-4-one (**1d**)

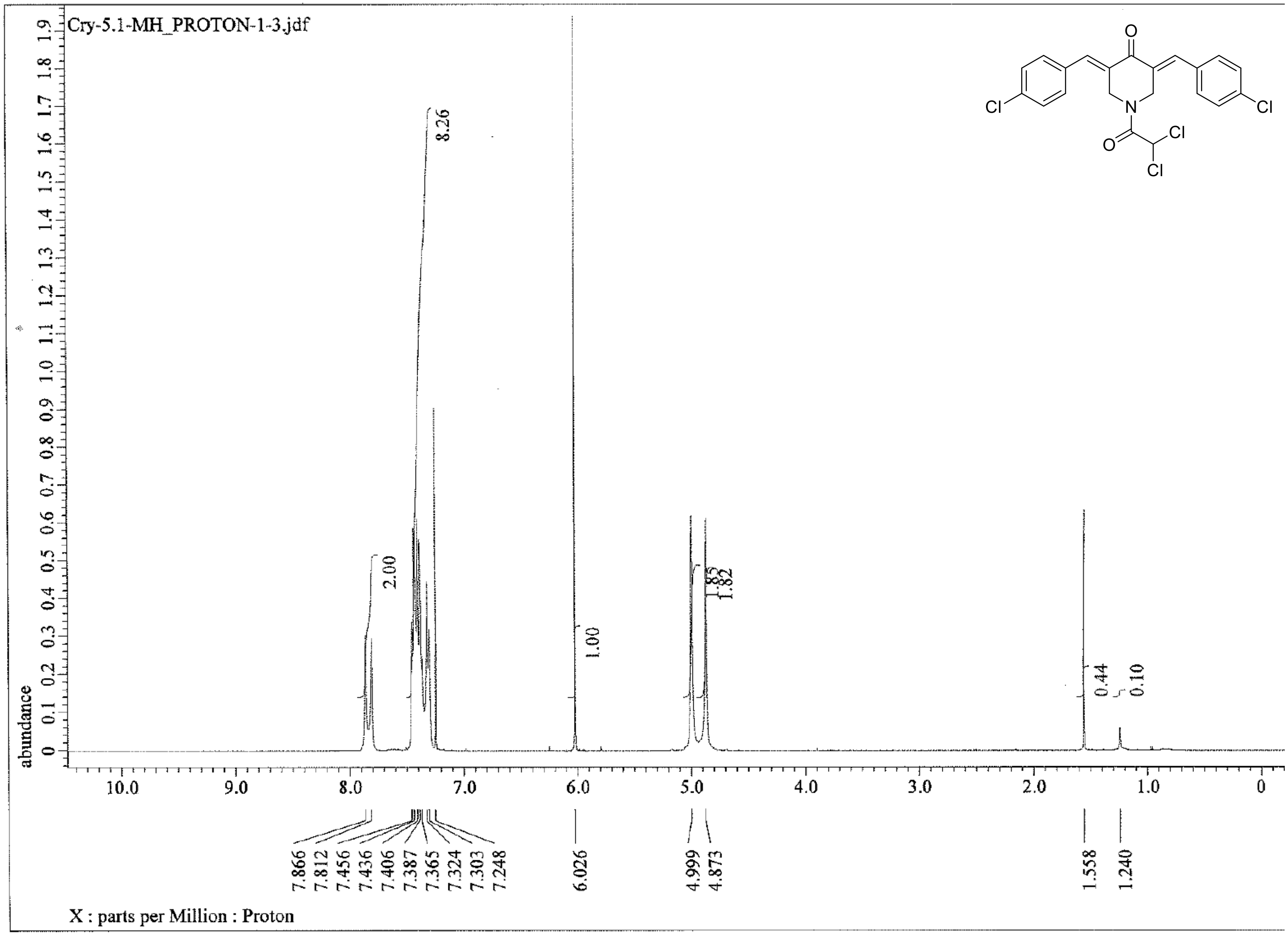

^{13}C NMR ($CDCl_3$) spectrum of 3,5-bis((*E*)-4-chlorobenzylidene)-1-(2,2-dichloroacetyl)piperidin-4-one (**1d**)

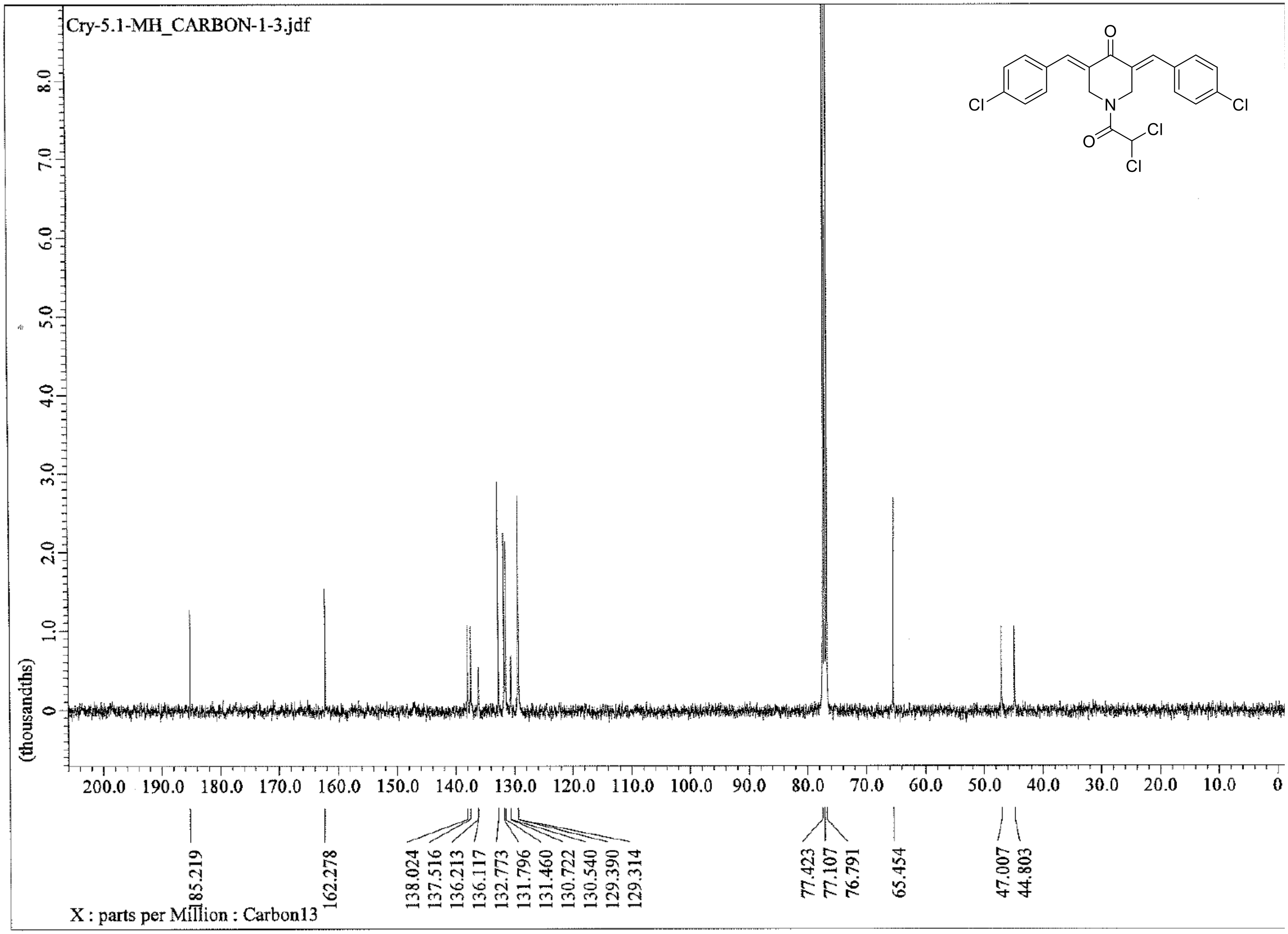

Mass spectrum of 3,5-bis((*E*)-4-chlorobenzylidene)-1-(2,2-dichloroacetyl)piperidin-4-one (**1d**)

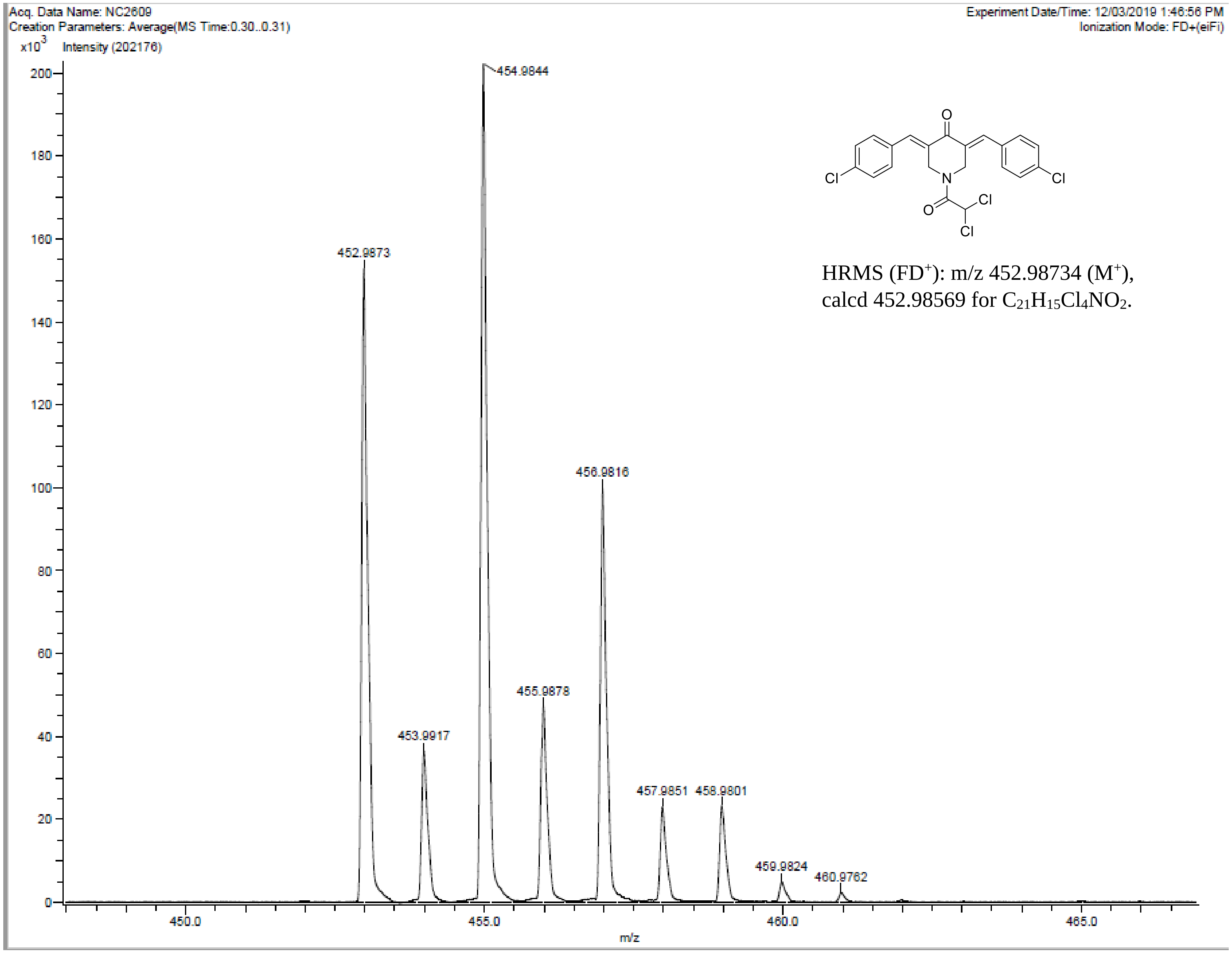

HRMS (FD$^+$): m/z 452.98734 (M$^+$), calcd 452.98569 for $C_{21}H_{15}Cl_4NO_2$.

^{1}H NMR ($CDCl_3$) spectrum of 1-(2,2-dichloroacetyl)-3,5-bis((*E*)-3,4-dichlorobenzylidene)piperidin-4-one (**1e**)

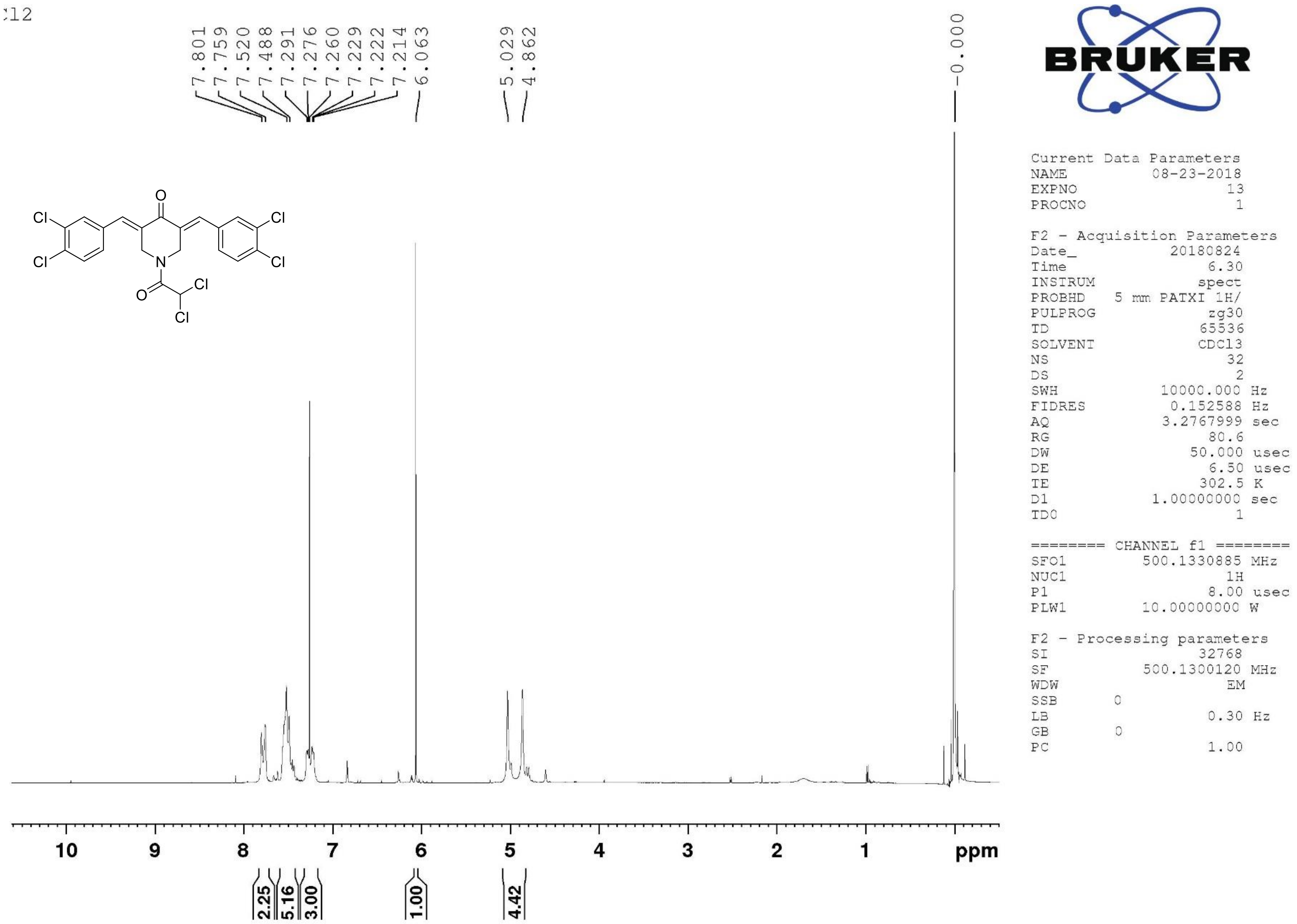

^{13}C NMR ($CDCl_3$) spectrum of 1-(2,2-dichloroacetyl)-3,5-bis((*E*)-3,4-dichlorobenzylidene)piperidin-4-one (**1e**)

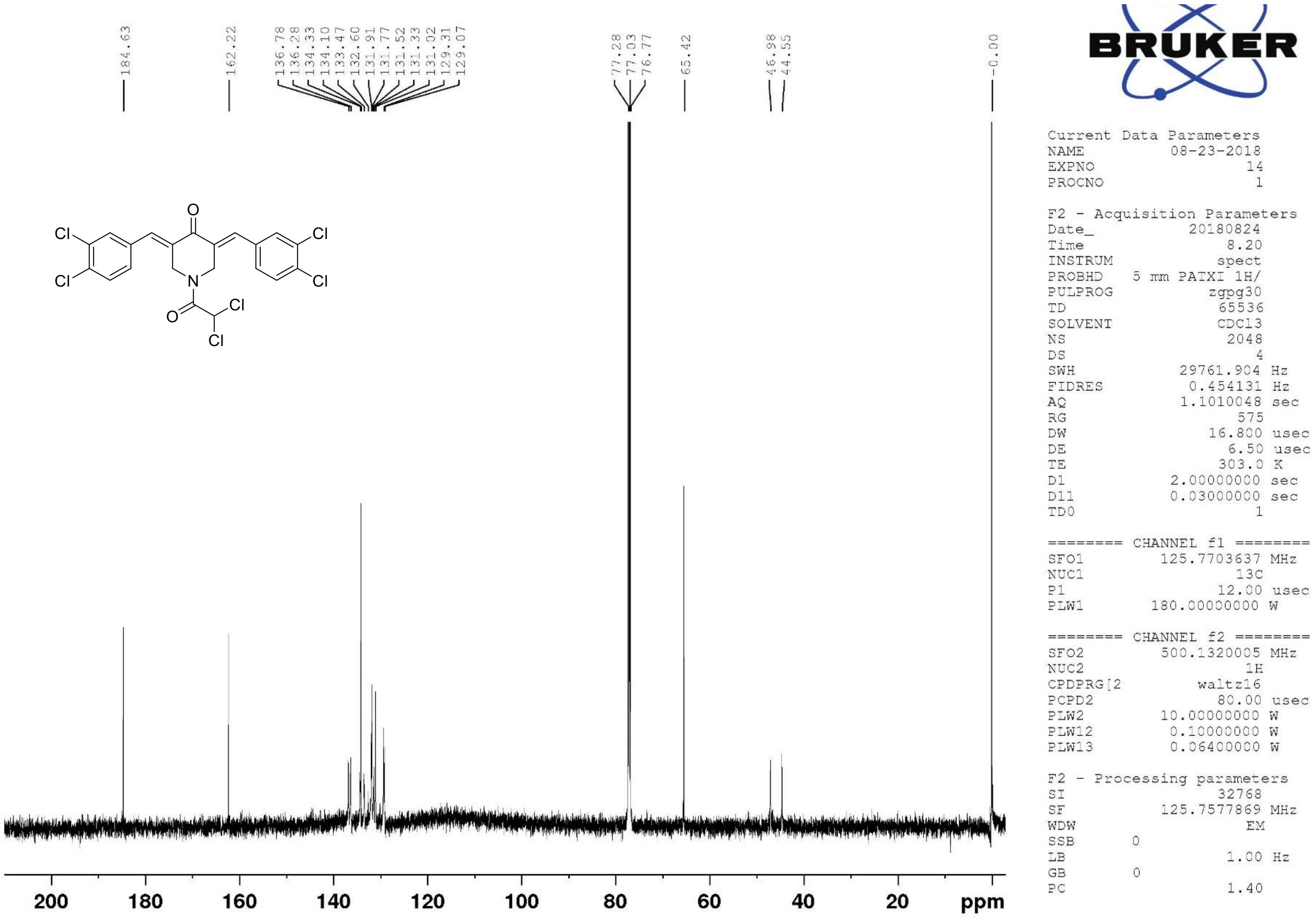

Mass spectrum of 1-(2,2-dichloroacetyl)-3,5-bis((*E*)-3,4-dichlorobenzylidene)piperidin-4-one (**1e**)

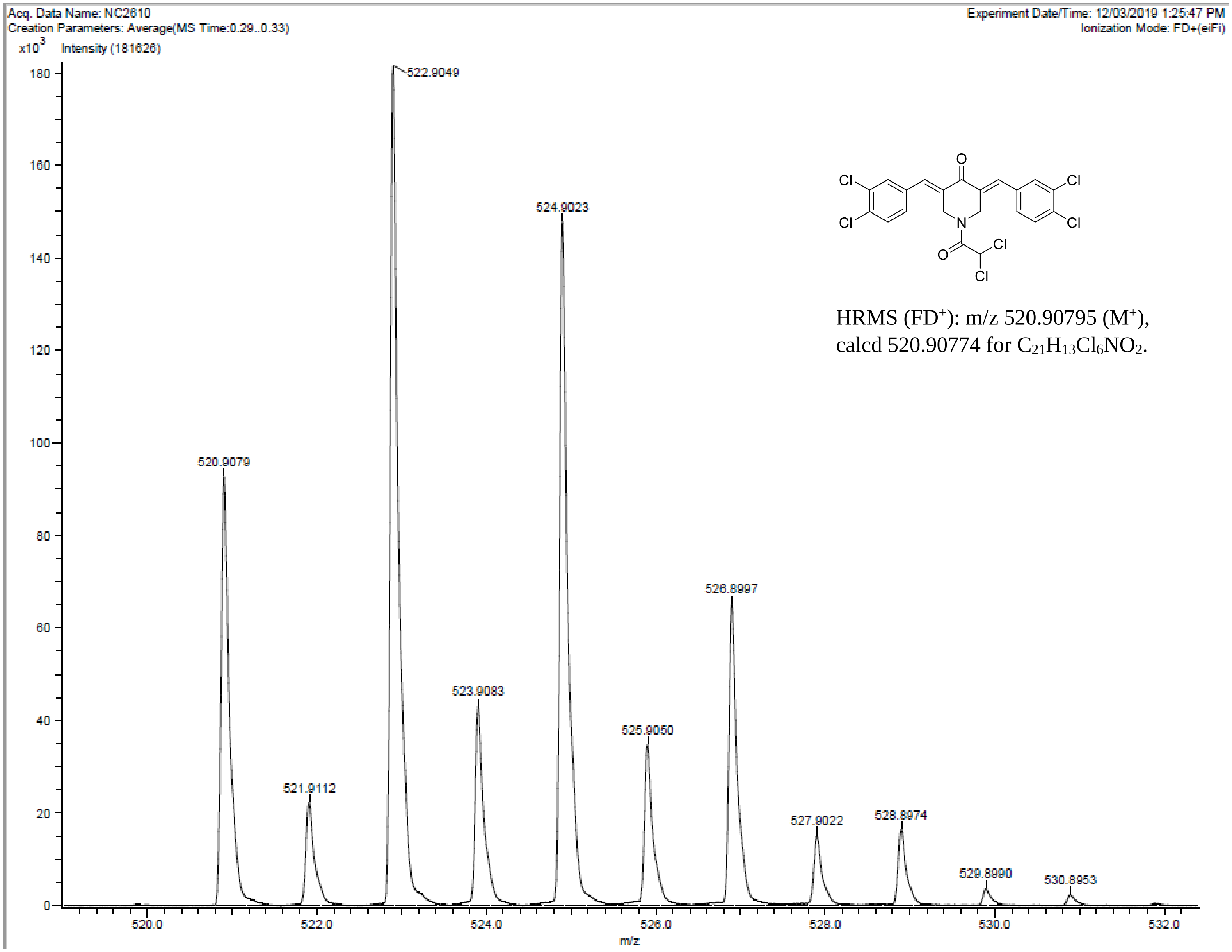

HRMS (FD^+): m/z 520.90795 (M^+), calcd 520.90774 for $C_{21}H_{13}Cl_6NO_2$.

^{1}H NMR ($CDCl_3$) spectrum of 1-(2,2-dichloroacetyl)-3,5-bis((*E*)-4-methoxybenzylidene)piperidin-4-one (**1f**)

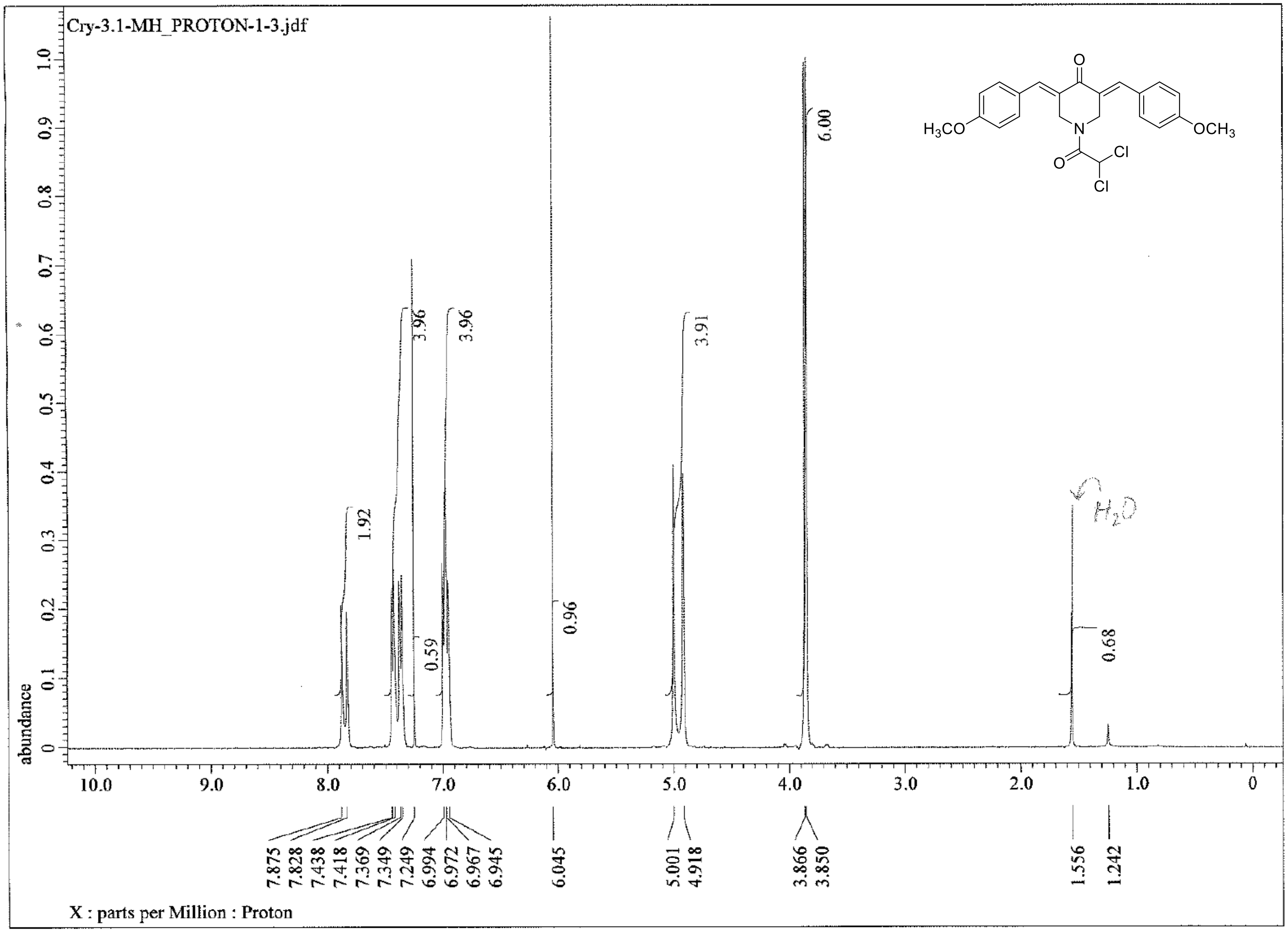

^{13}C NMR ($CDCl_3$) spectrum of 1-(2,2-dichloroacetyl)-3,5-bis((*E*)-4-methoxybenzylidene)piperidin-4-one (**1f**)

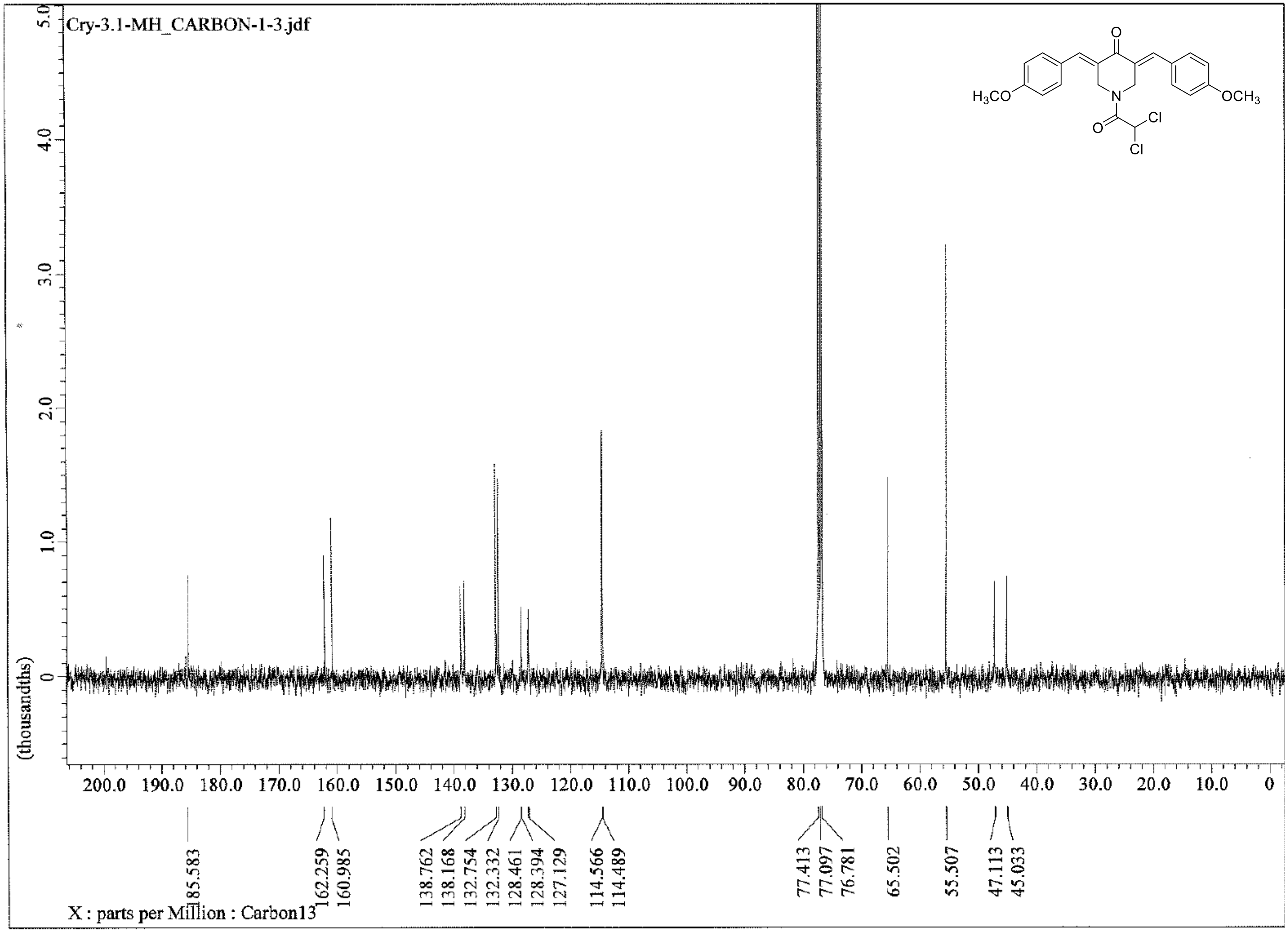

Mass spectrum of 1-(2,2-dichloroacetyl)-3,5-bis((*E*)-4-methoxybenzylidene)piperidin-4-one (**1f**)

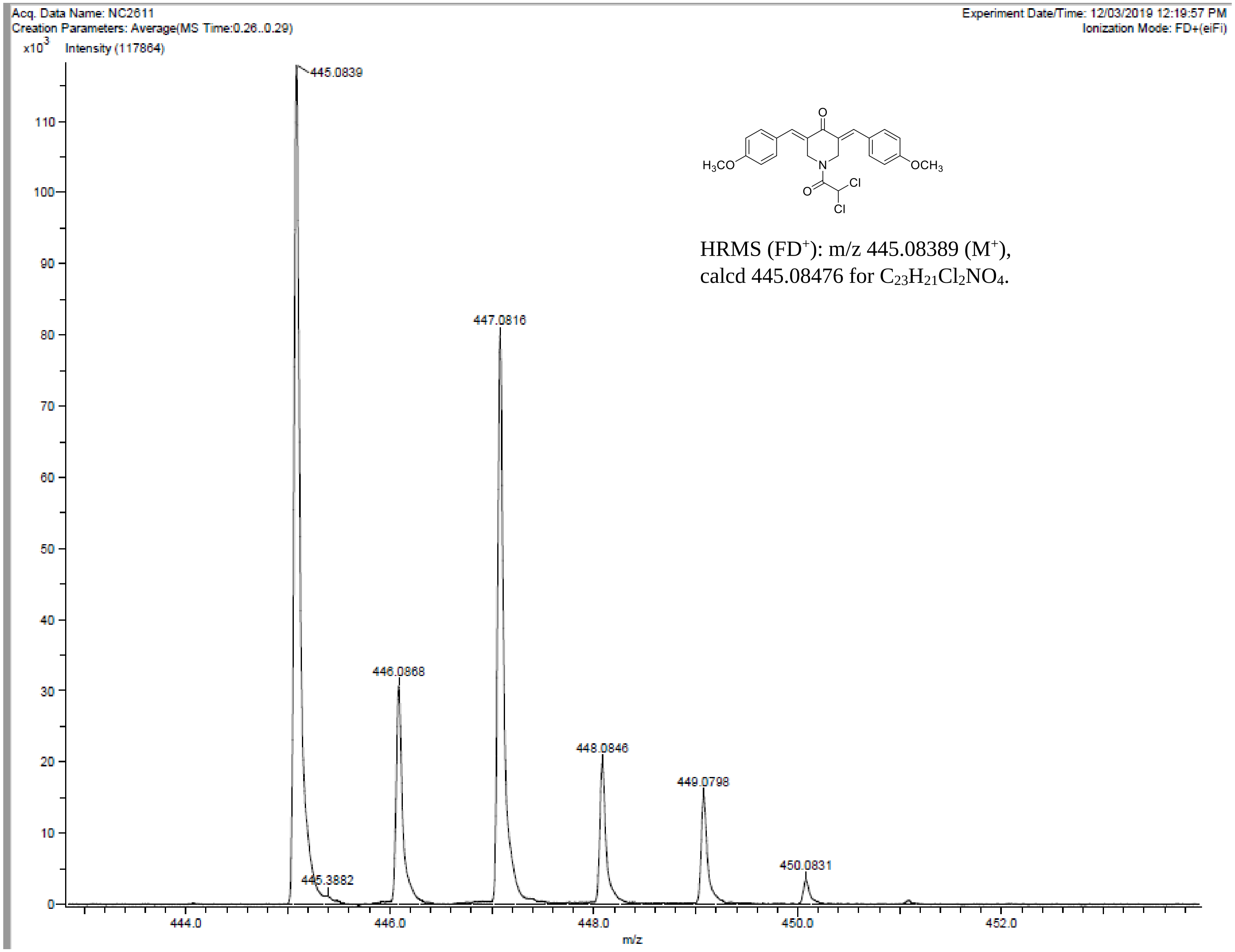

^{1}H NMR ($(CD_3)_2SO$) spectrum of 1-(2,2-dichloroacetyl)-3,5-bis((*E*)-3,4-dimethoxybenzylidene)piperidin-4-one (**1g**)

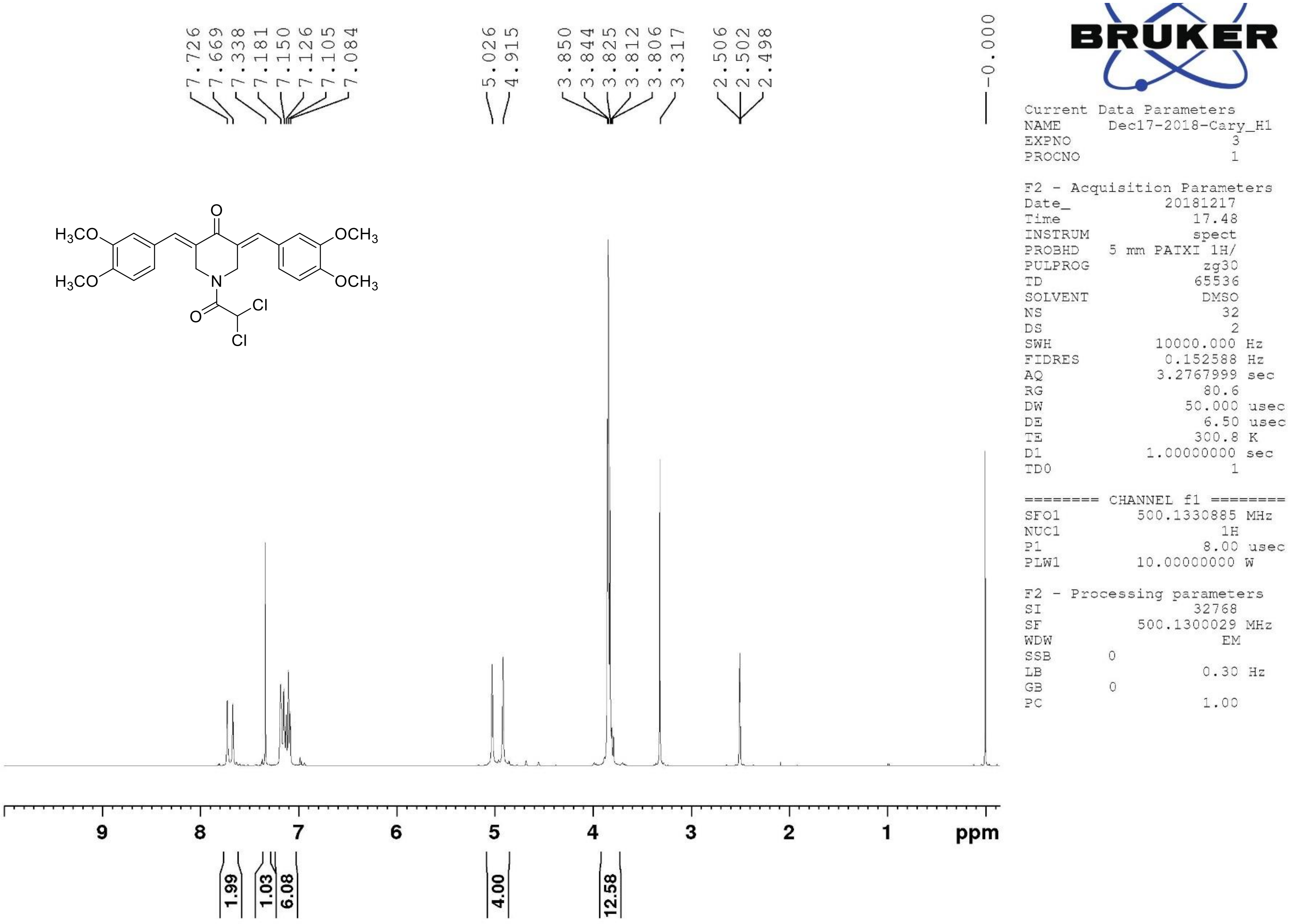

^{13}C NMR ($(CD_3)_2SO$) spectrum of 1-(2,2-dichloroacetyl)-3,5-bis((*E*)-3,4-dimethoxybenzylidene)piperidin-4-one (**1g**)

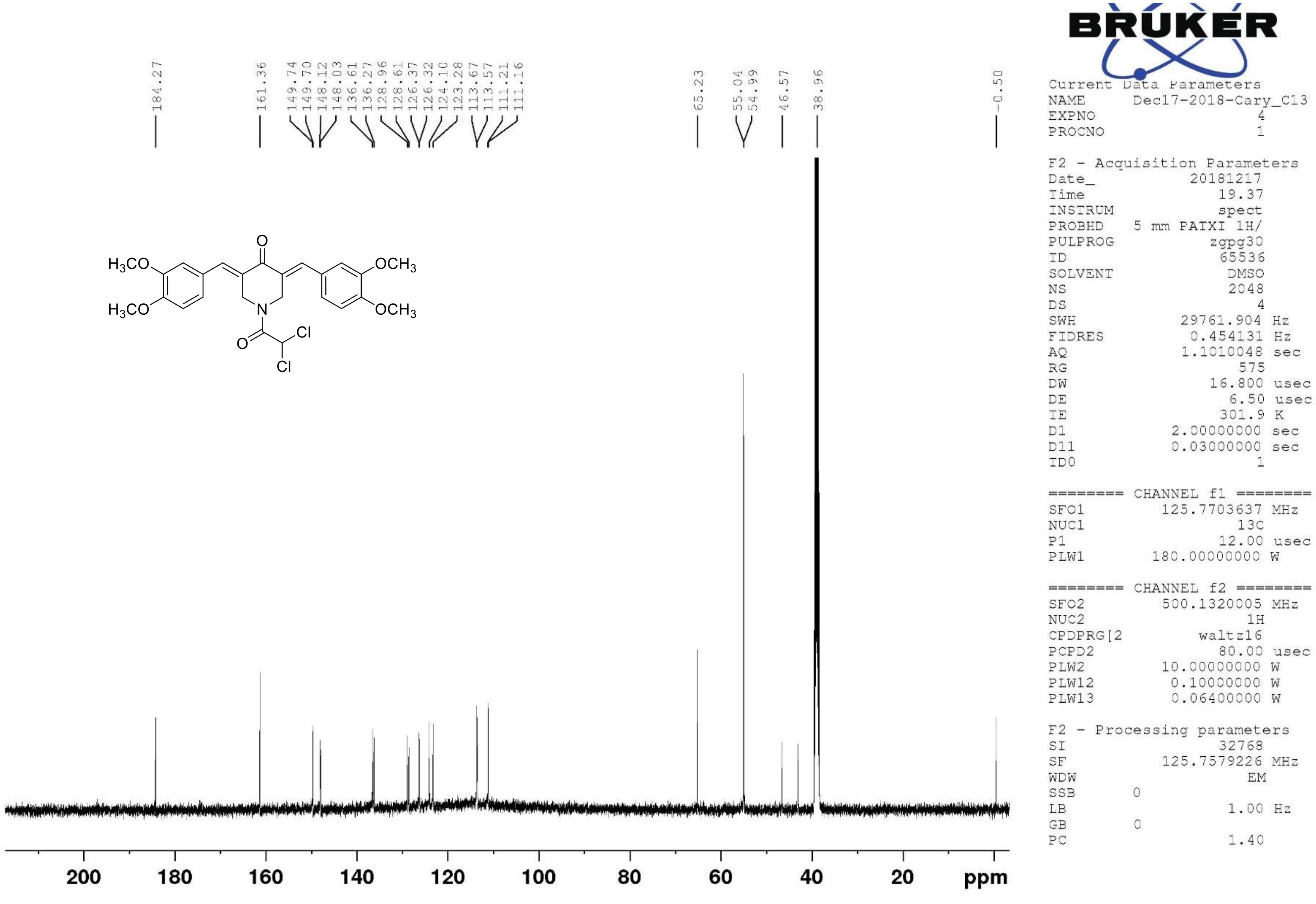

Mass spectrum of 1-(2,2-dichloroacetyl)-3,5-bis((*E*)-3,4-dimethoxybenzylidene)piperidin-4-one (**1g**)

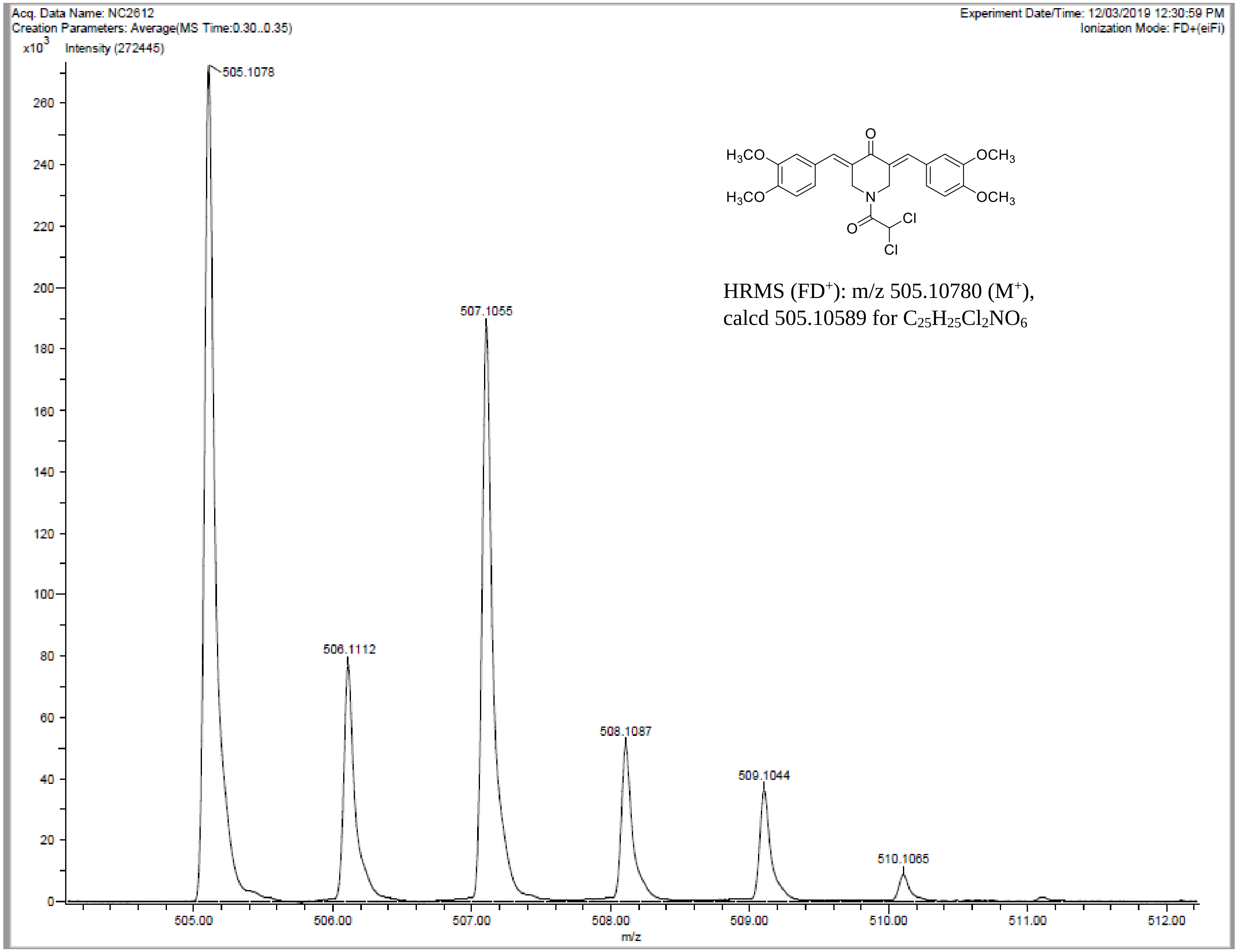

^{1}H NMR ($(CD_3)_2SO$) spectrum of 1-(2,2-dichloroacetyl)-3,5-bis((*E*)-3,4,5-trimethoxybenzylidene)piperidin-4-one (**1h**)

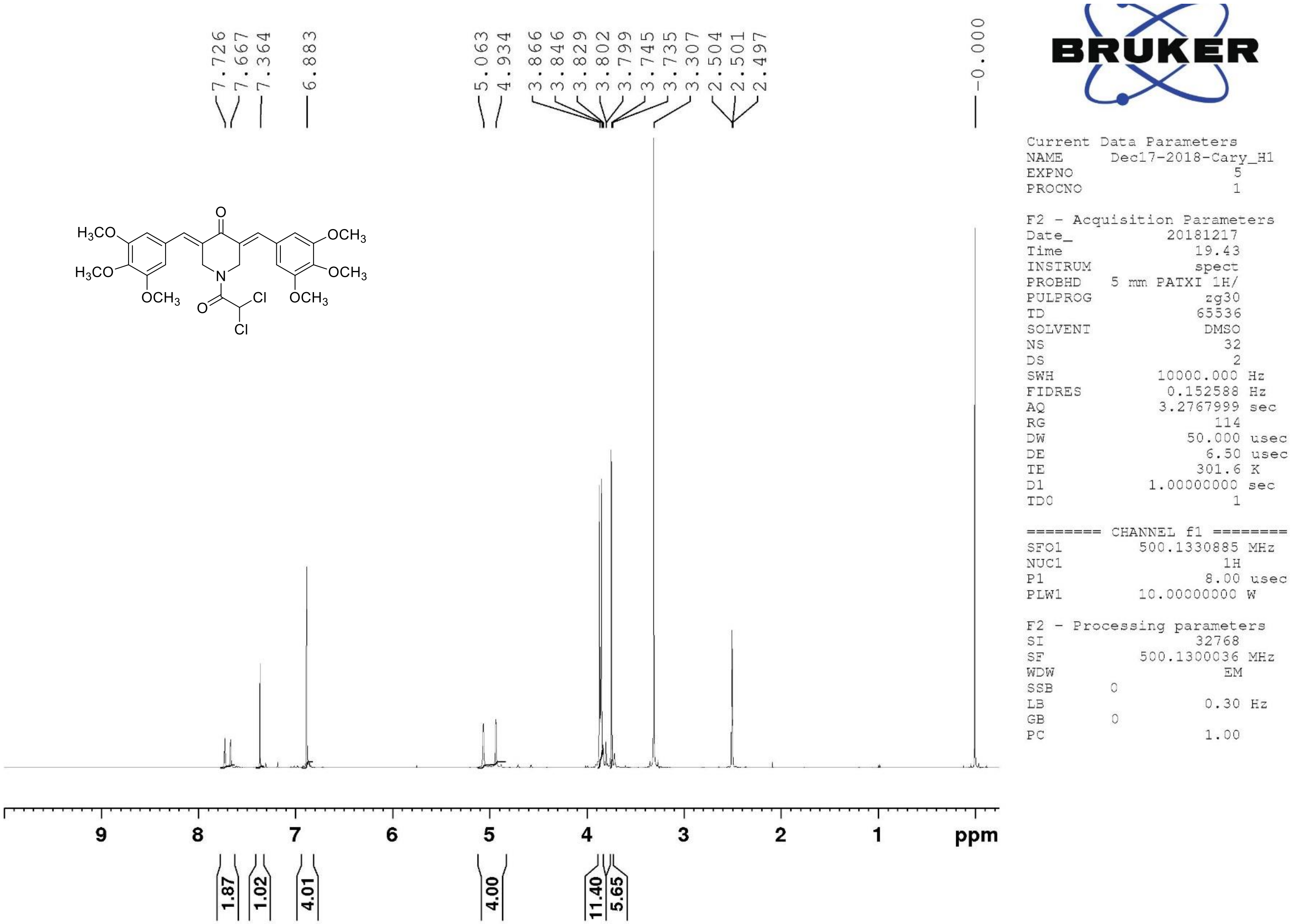

^{13}C NMR ((CD_3)$_2$SO) spectrum of 1-(2,2-dichloroacetyl)-3,5-bis((*E*)-3,4,5-trimethoxybenzylidene)piperidin-4-one (**1h**)

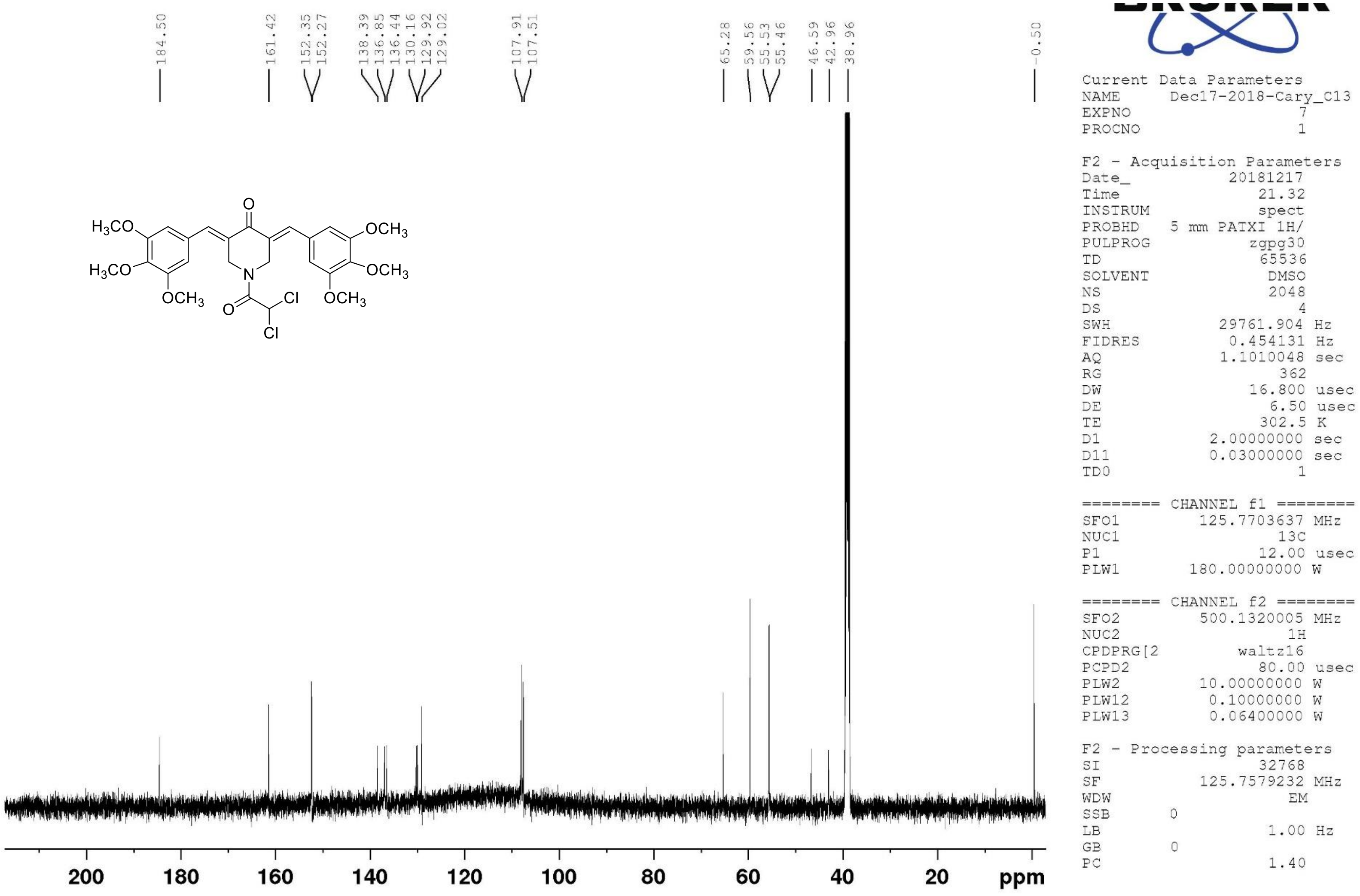

Mass spectrum of 1-(2,2-dichloroacetyl)-3,5-bis((*E*)-3,4,5-trimethoxybenzylidene)piperidin-4-one (**1h**)

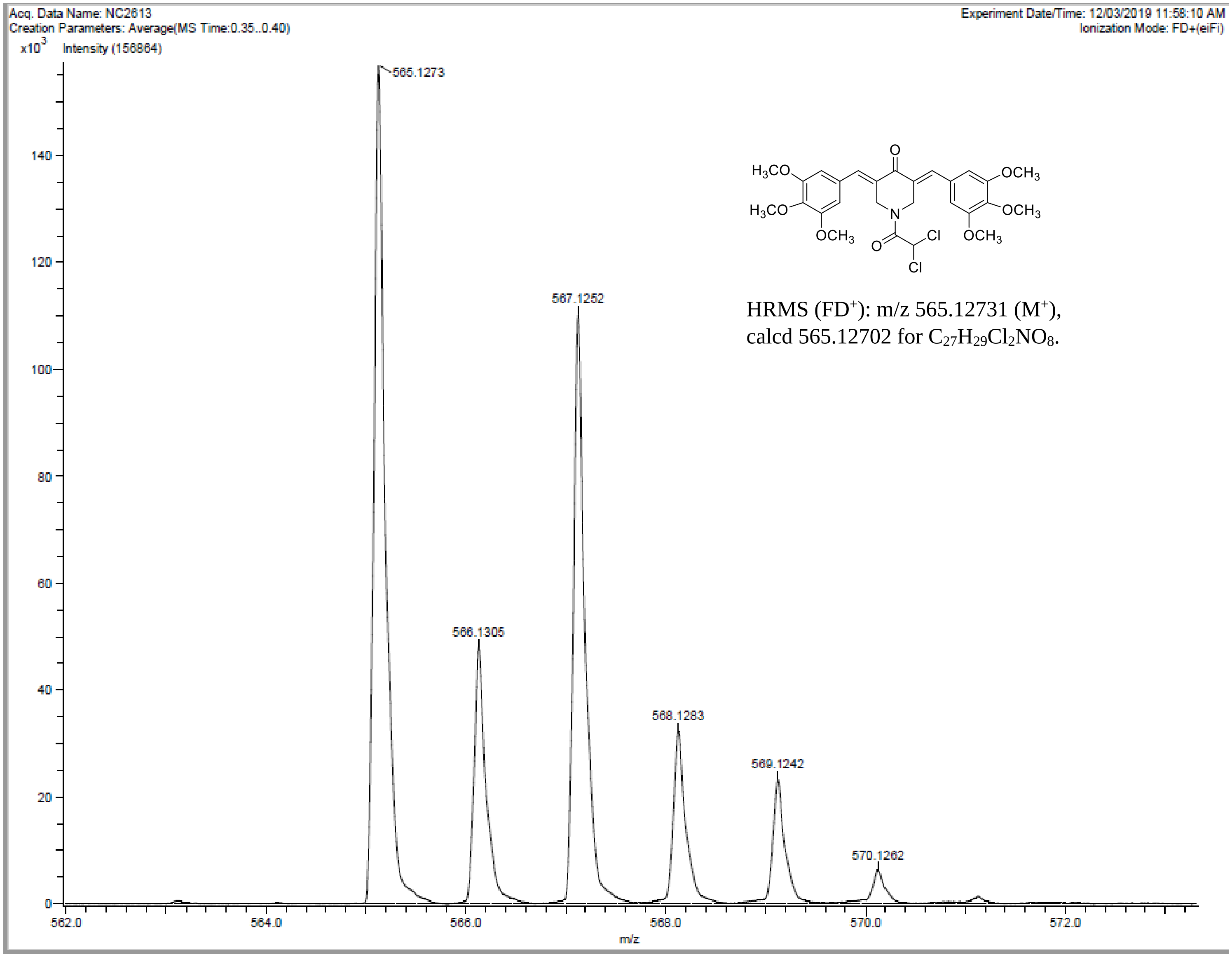

HRMS (FD$^+$): m/z 565.12731 (M$^+$), calcd 565.12702 for $C_{27}H_{29}Cl_2NO_8$.

^{1}H NMR ($(CD_3)_2SO$) spectrum of 1-(2,2-dichloroacetyl)-3,5-bis((*E*)-3,4-methylenedioxybenzylidene)piperidin-4-one (**1i**)

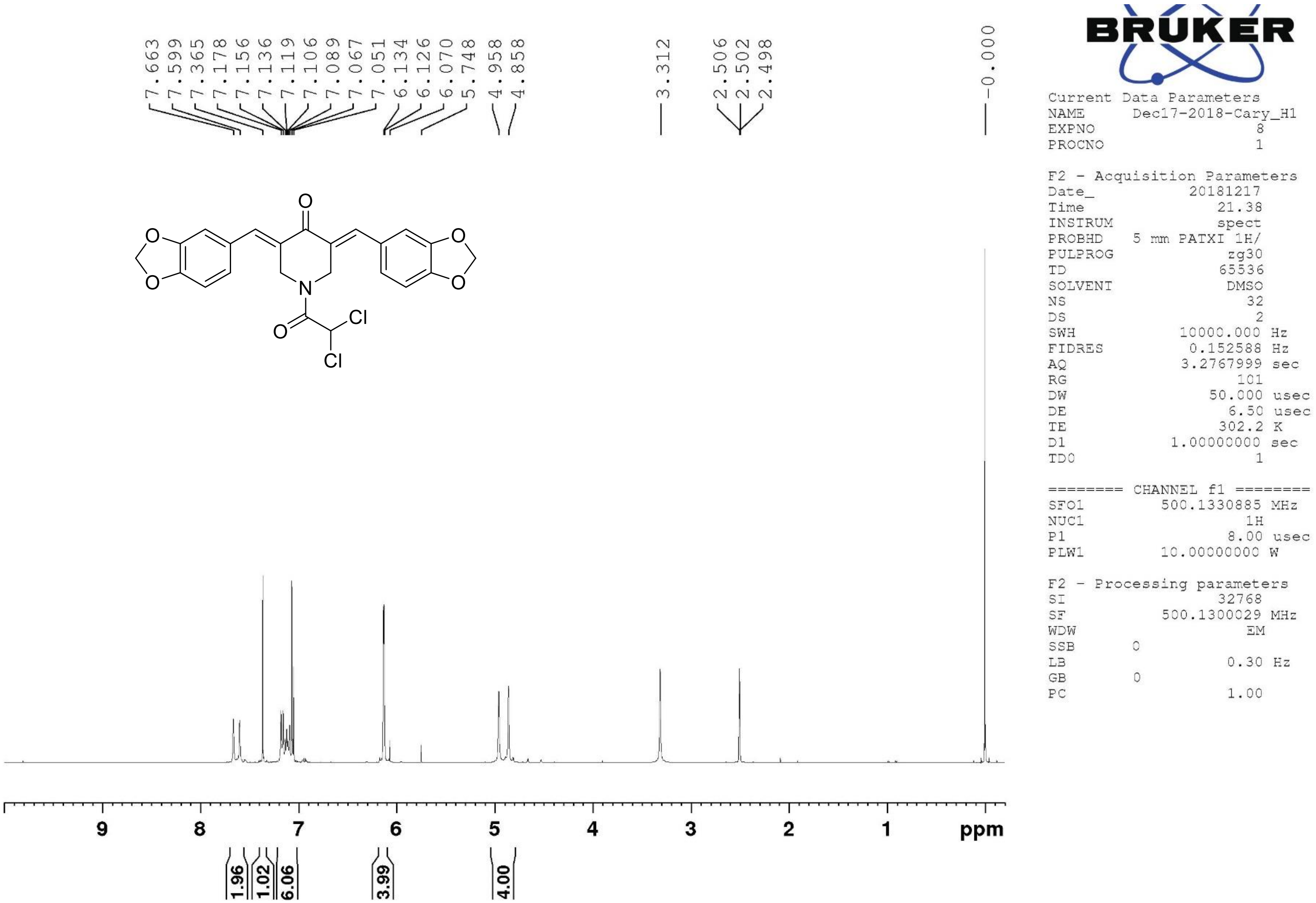

^{13}C NMR ($(CD_3)_2SO$) spectrum of 1-(2,2-dichloroacetyl)-3,5-bis((*E*)-3,4-methylenedioxybenzylidene)piperidin-4-one (**1i**)

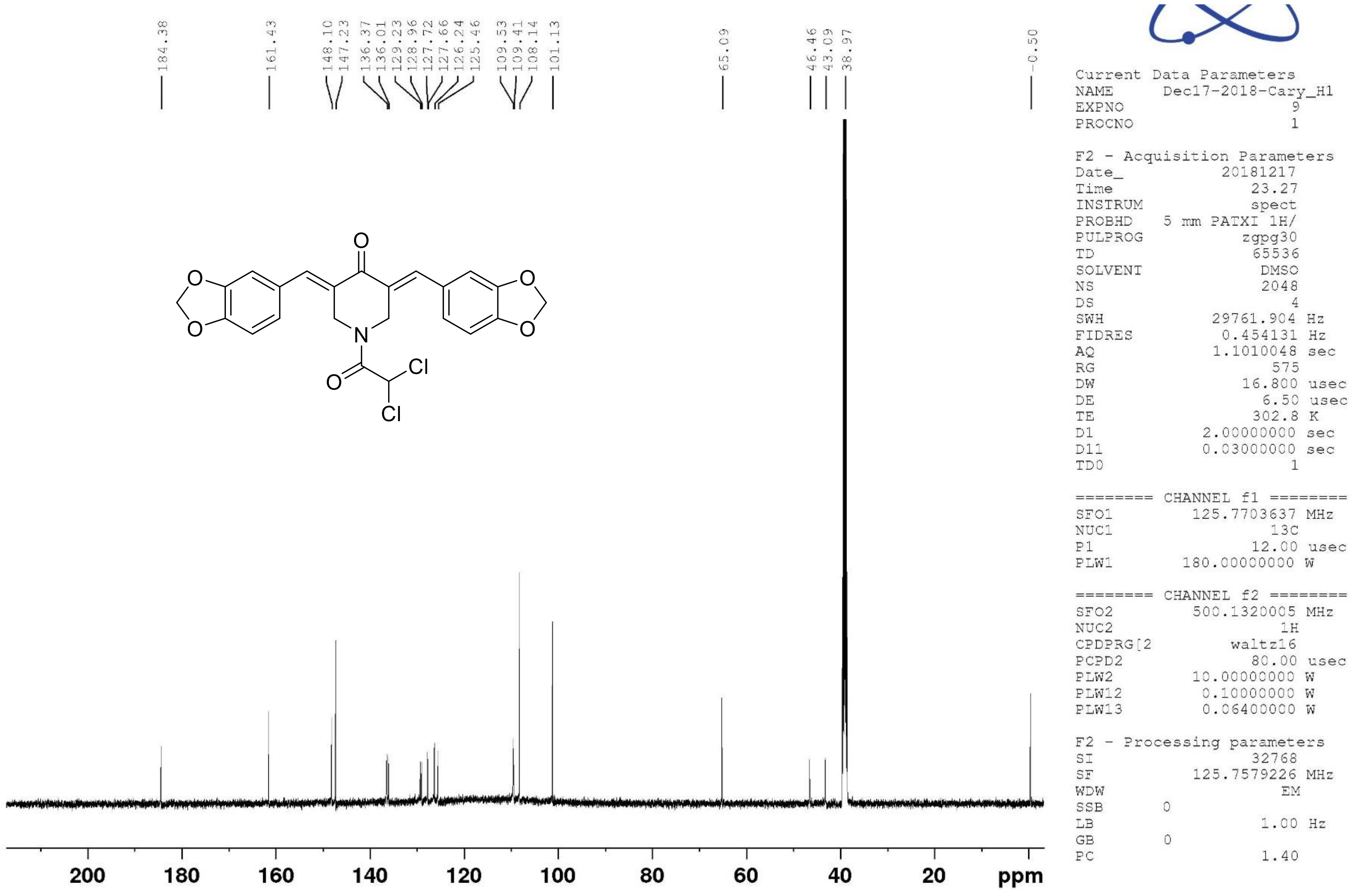

Mass spectrum of 1-(2,2-dichloroacetyl)-3,5-bis((*E*)-3,4-methylenedioxybenzylidene)piperidin-4-one (**1i**)

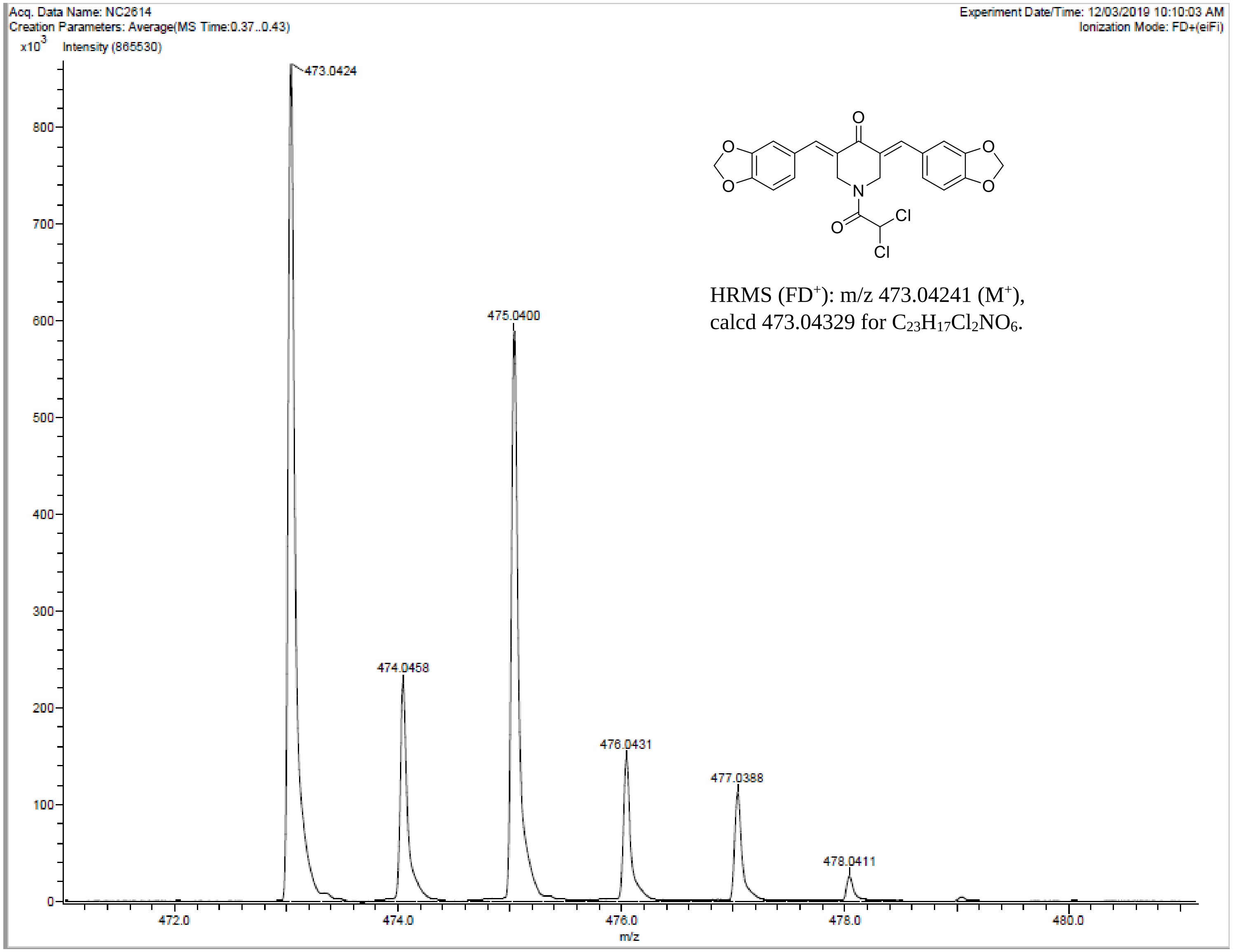

^{1}H NMR ($CDCl_3$) spectrum of 1-(2,2-dichloroacetyl)-3,5-bis((*E*)-4-methylbenzylidene)piperidin-4-one (**1j**)

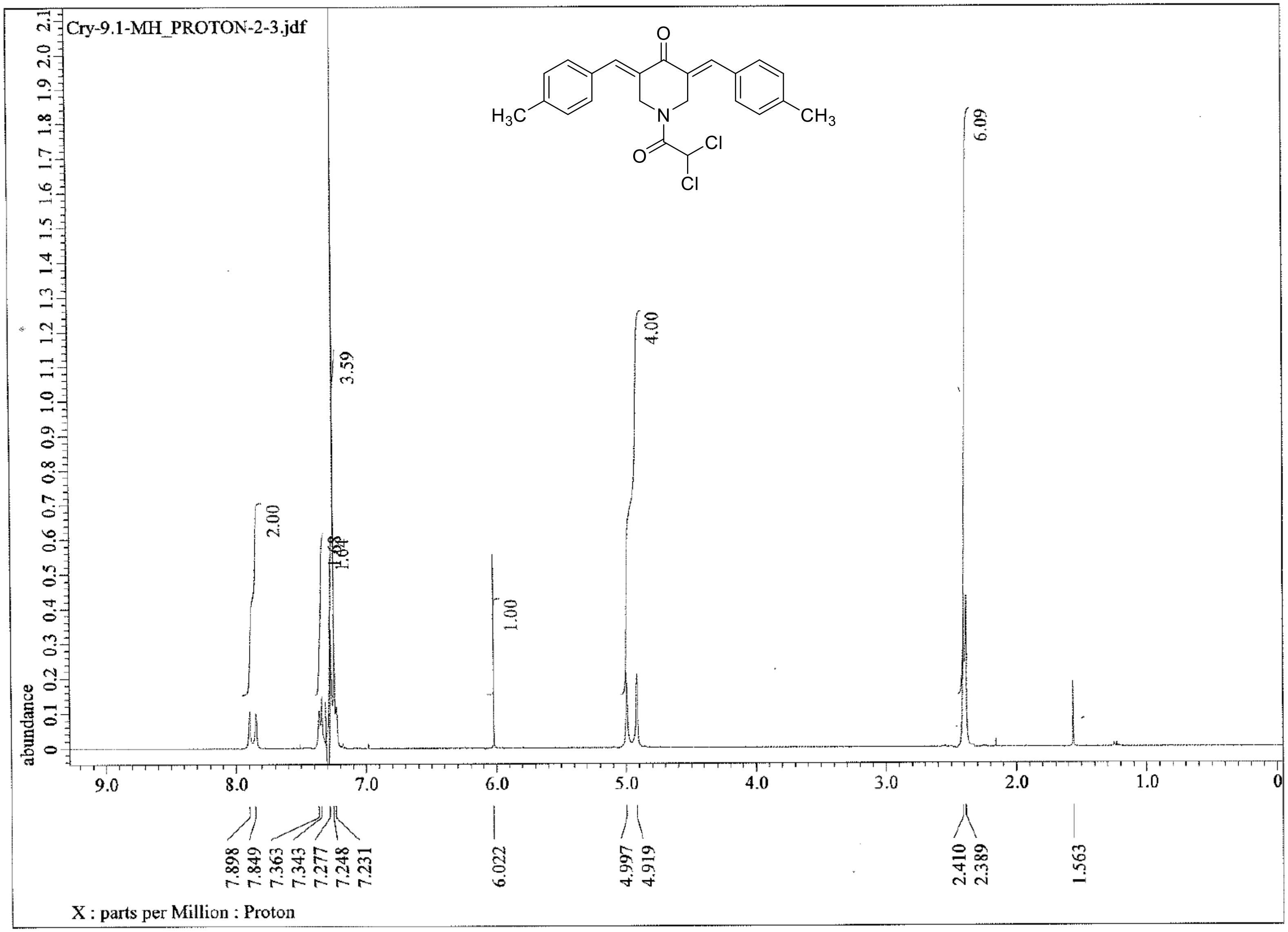

^{13}C NMR ($CDCl_3$) spectrum of 1-(2,2-dichloroacetyl)-3,5-bis((*E*)-4-methylbenzylidene)piperidin-4-one (**1j**)

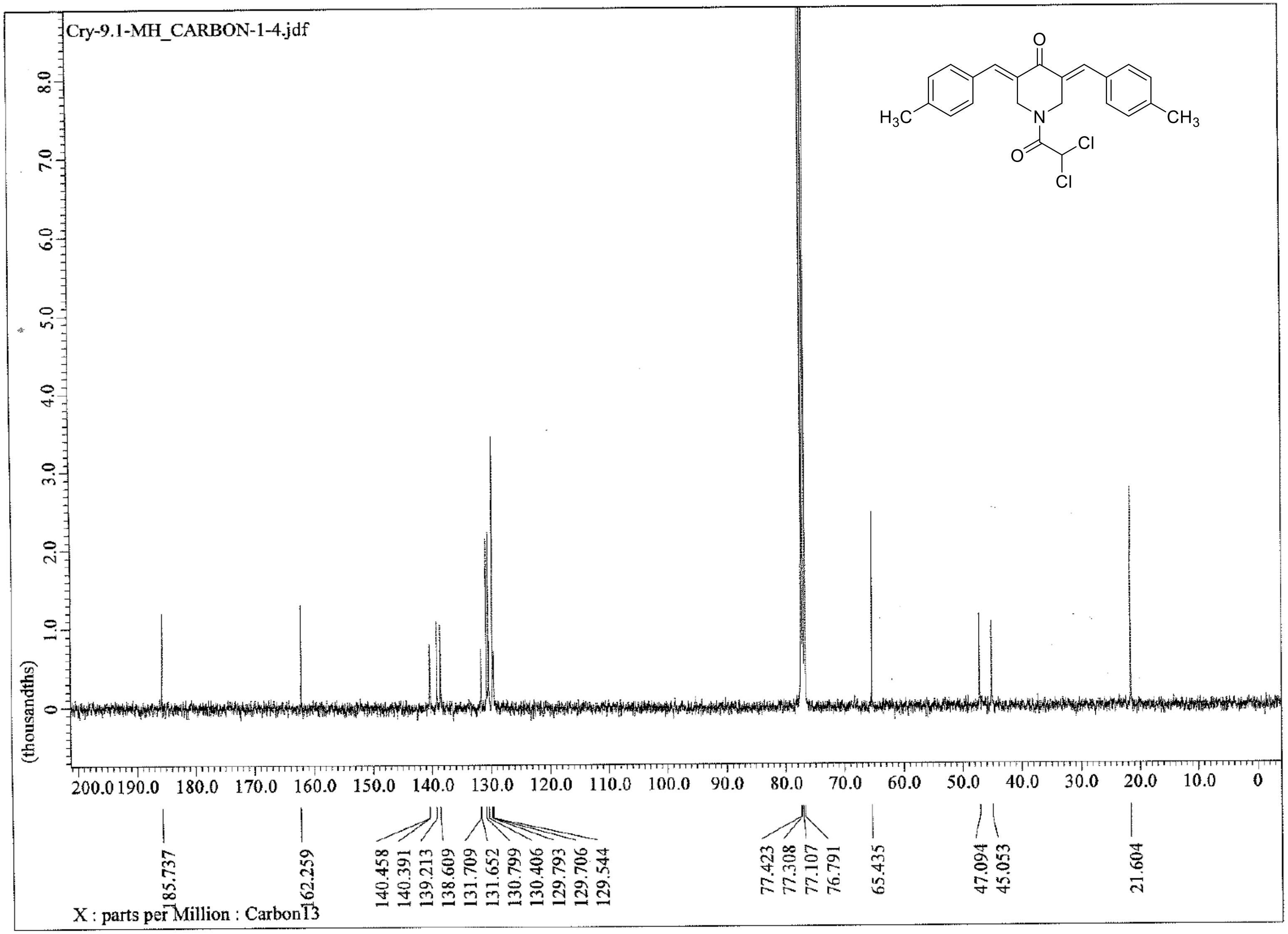

Mass spectrum of 1-(2,2-dichloroacetyl)-3,5-bis((*E*)-4-methylbenzylidene)piperidin-4-one (**1j**)

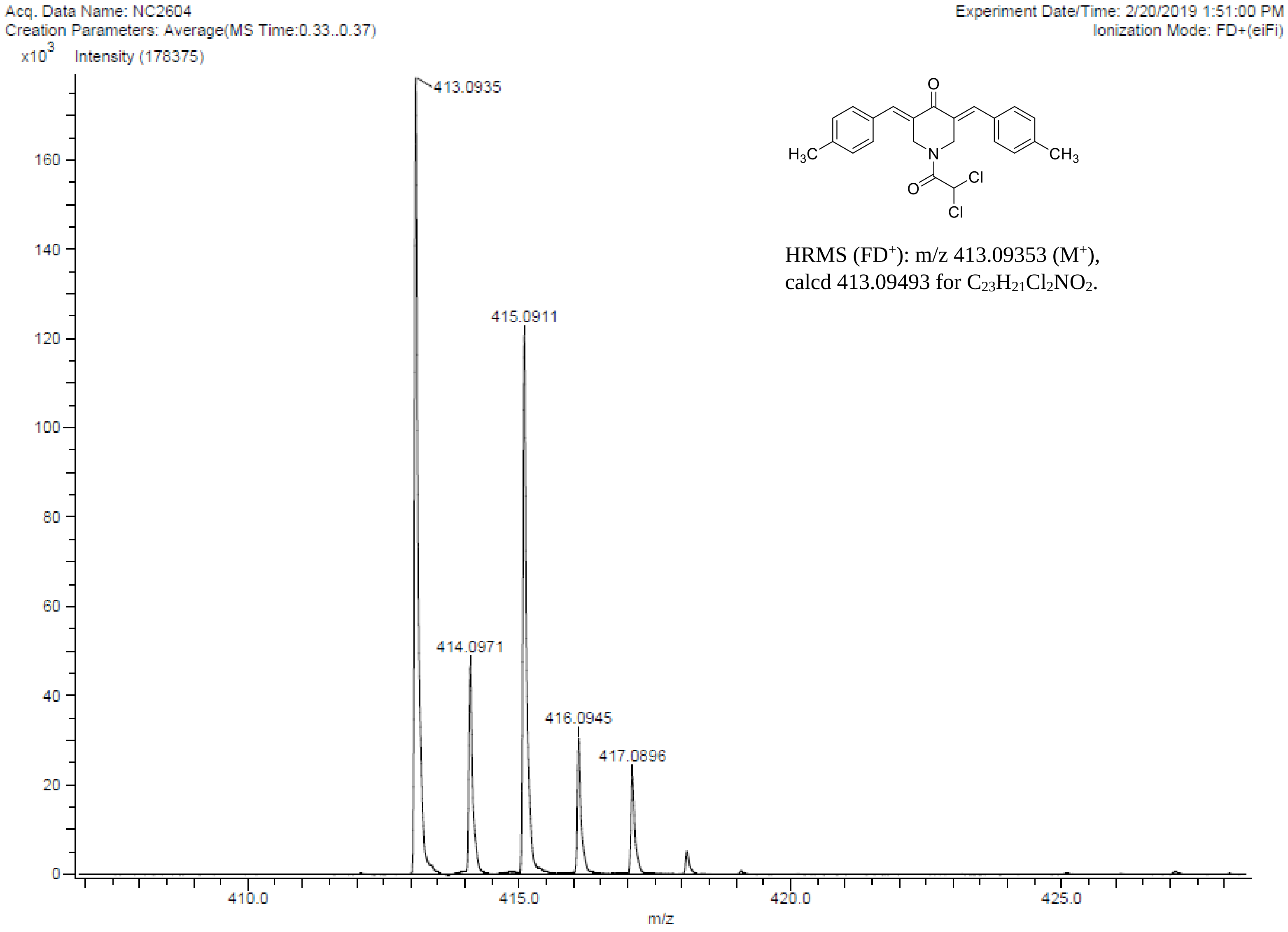

HRMS (FD$^+$): m/z 413.09353 (M$^+$), calcd 413.09493 for $C_{23}H_{21}Cl_2NO_2$.

^{1}H NMR ($(CD_3)_2SO$) spectrum of 1-(2,2-dichloroacetyl)-3,5-bis((*E*)-4-nitrobenzylidene)piperidin-4-one (**1k**)

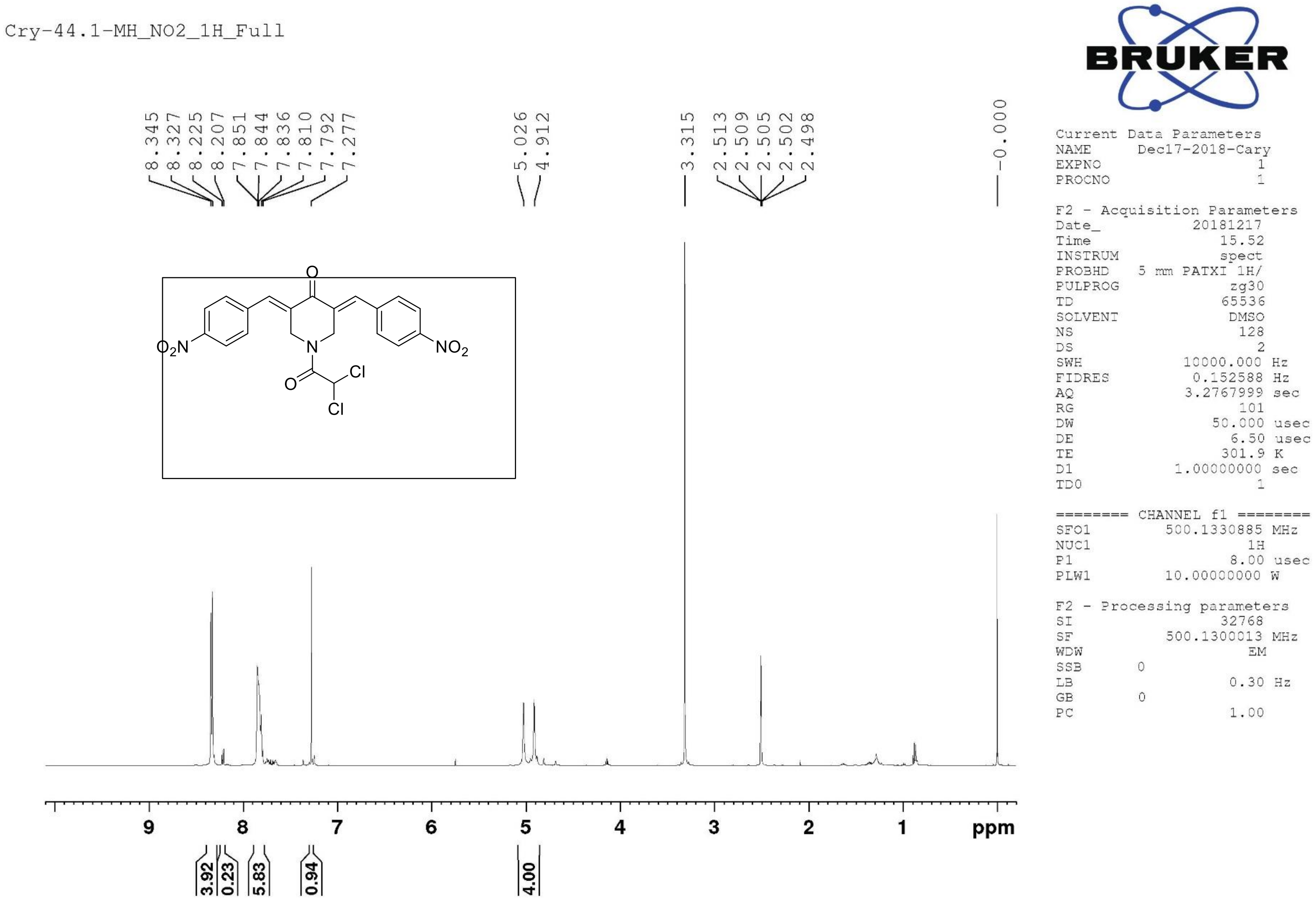

^{13}C NMR ($(CD_3)_2SO$) spectrum of 1-(2,2-dichloroacetyl)-3,5-bis((*E*)-4-nitrobenzylidene)piperidin-4-one (**1k**)

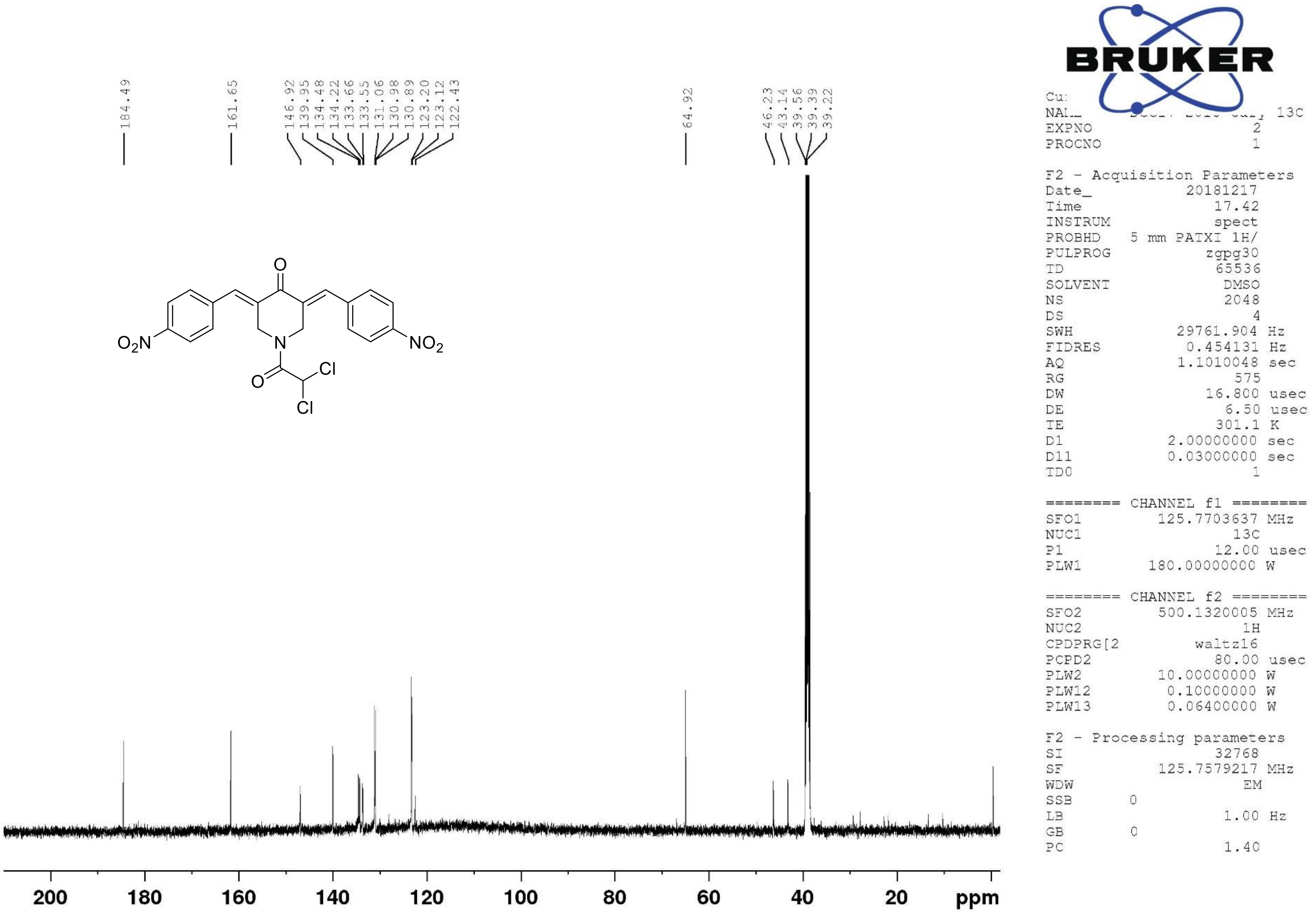

Mass spectrum of 1-(2,2-dichloroacetyl)-3,5-bis((*E*)-4-nitrobenzylidene)piperidin-4-one (**1k**)

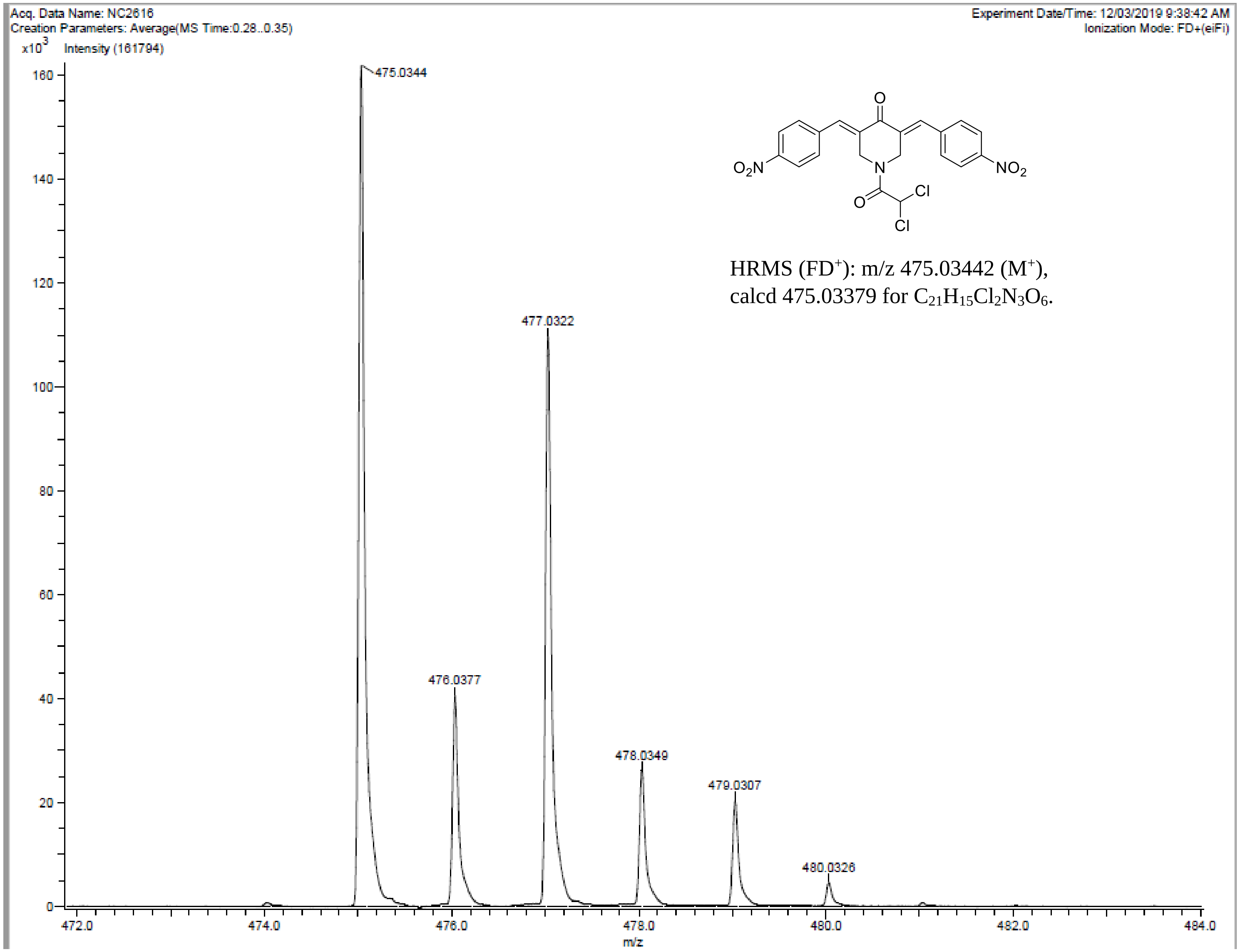

HRMS (FD$^+$): m/z 475.03442 (M$^+$), calcd 475.03379 for $C_{21}H_{15}Cl_2N_3O_6$.

^{1}H NMR ($CDCl_3$) spectrum of 1-(2,2-dichloroacetyl)-3,5-bis((*E*)-4-(dimethylamino)benzylidene)piperidin-4-one (**1l**)

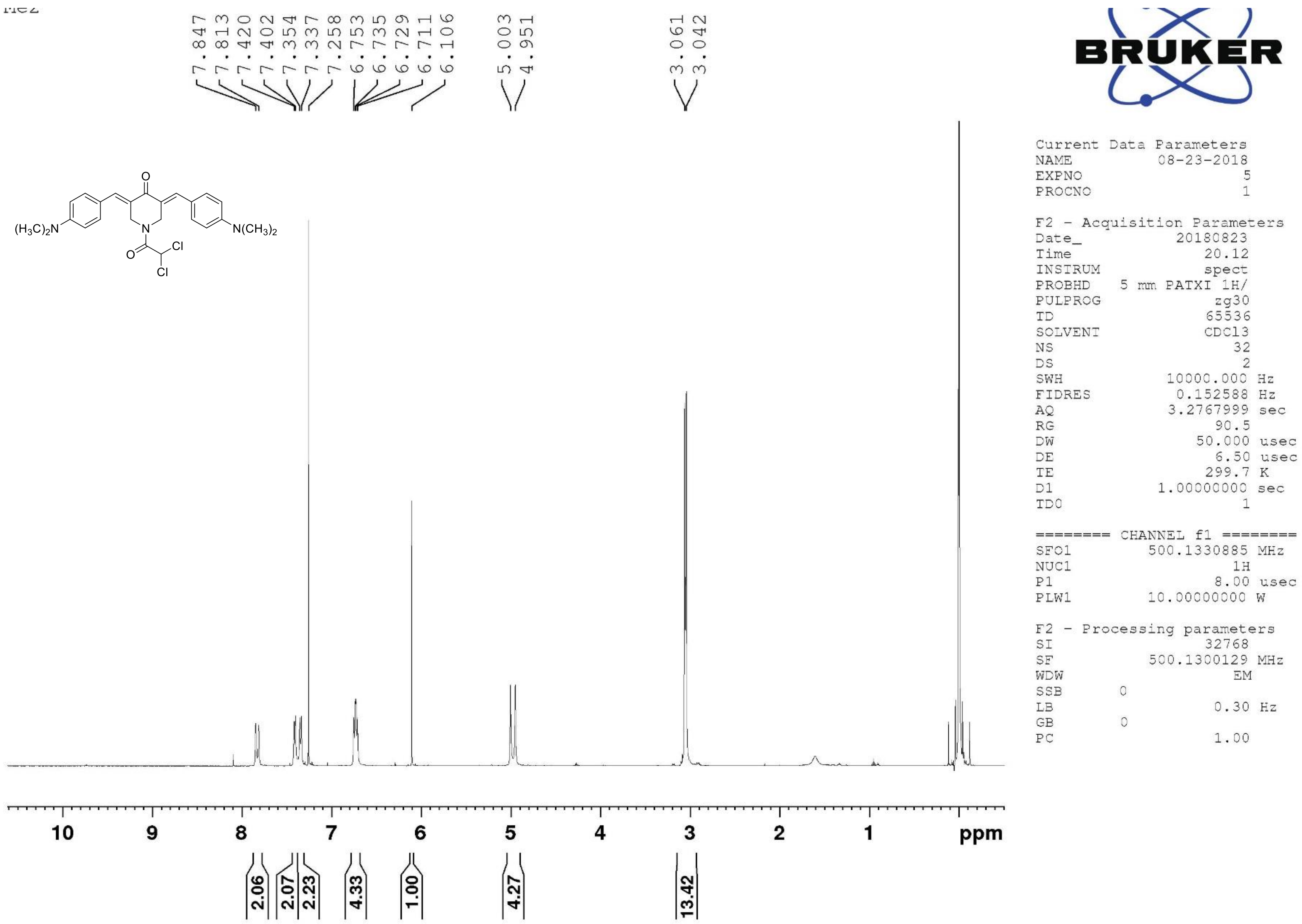

^{13}C NMR ($CDCl_3$) spectrum of 1-(2,2-dichloroacetyl)-3,5-bis((*E*)-4-(dimethylamino)benzylidene)piperidin-4-one (**1l**)

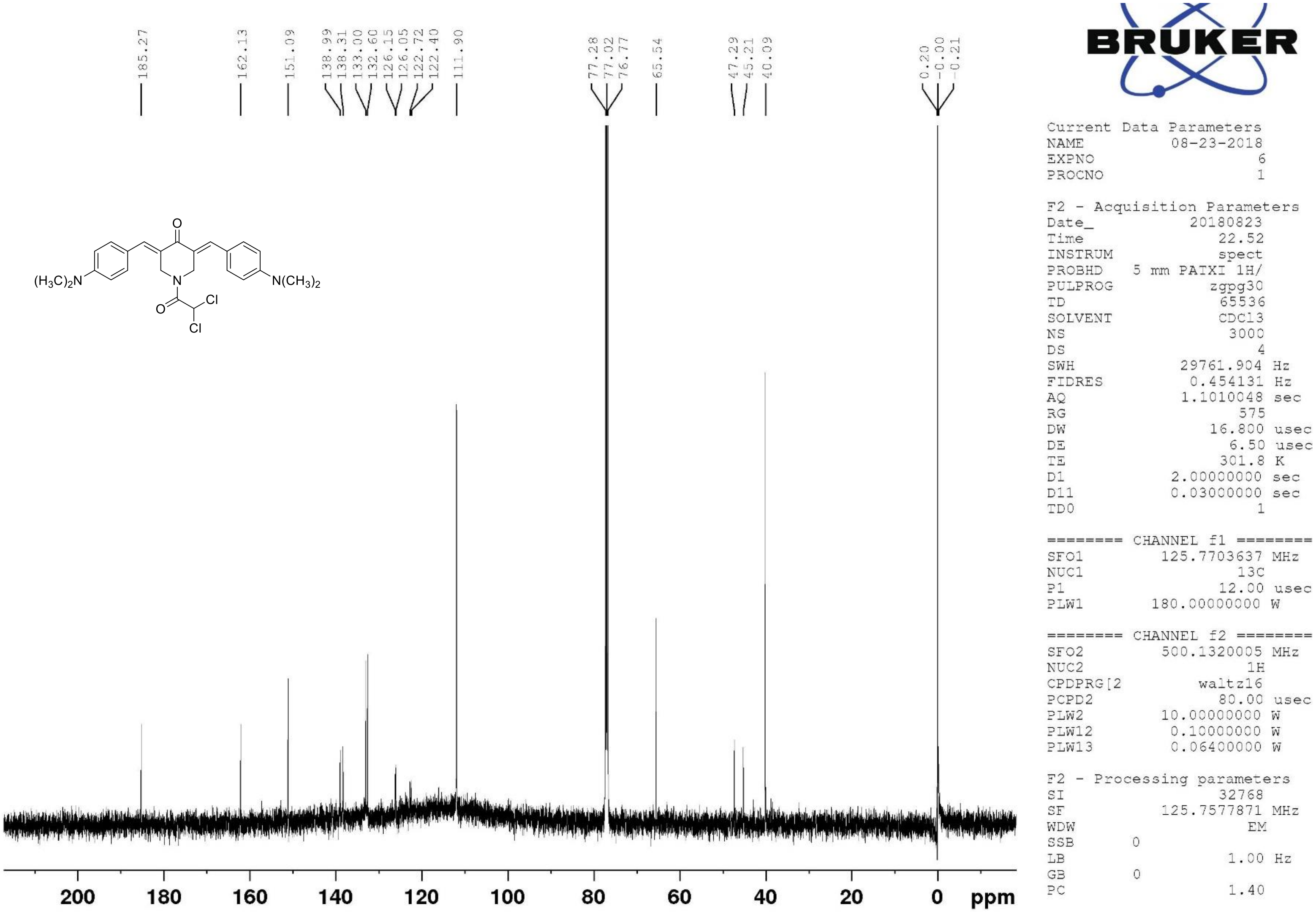

Mass spectrum of 1-(2,2-dichloroacetyl)-3,5-bis((E)-4-(dimethylamino)benzylidene)piperidin-4-one (**1l**)

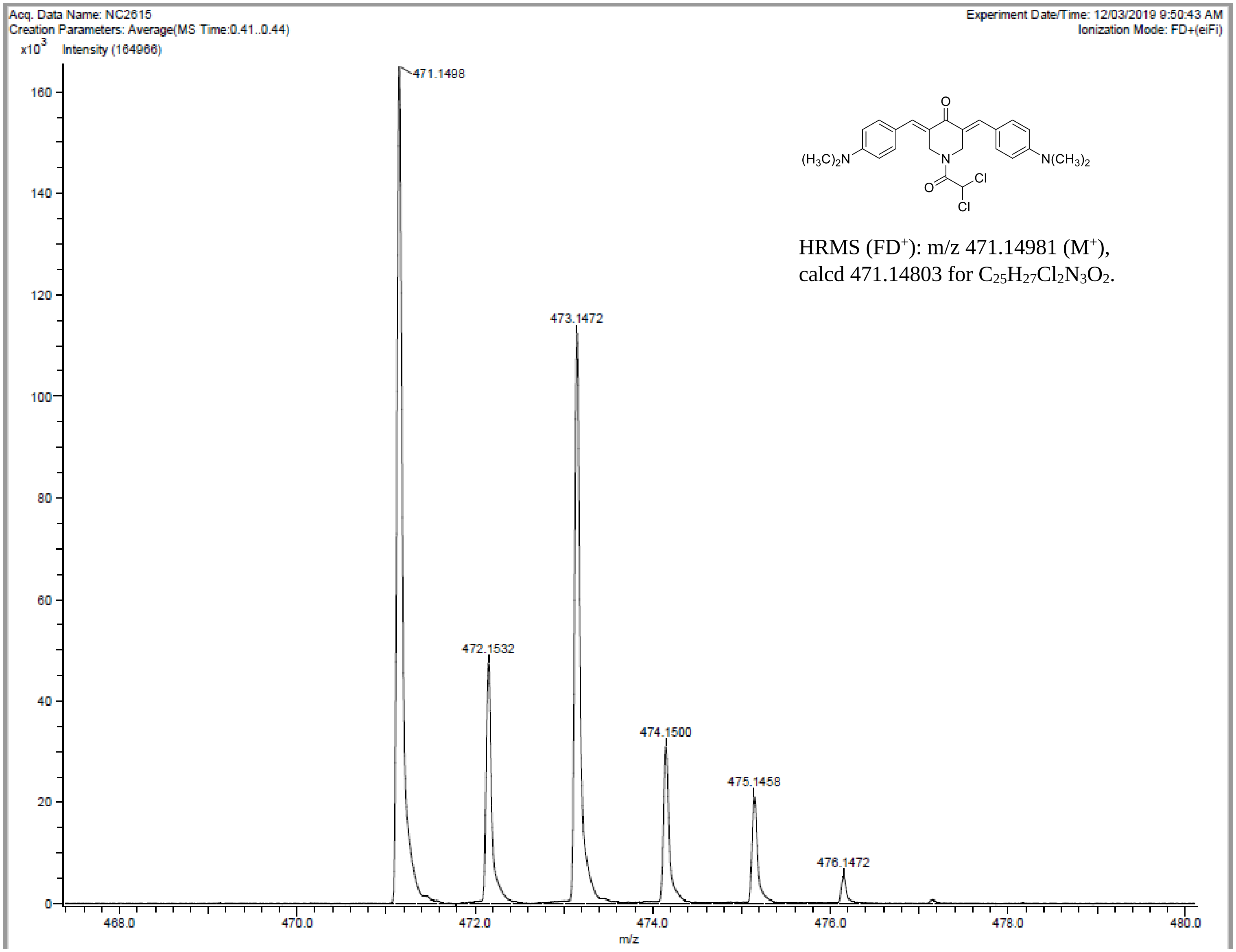

HRMS (FD^+): m/z 471.14981 (M^+), calcd 471.14803 for $C_{25}H_{27}Cl_2N_3O_2$.

^{1}H NMR ($(CD_3)_2SO$) spectrum of 3,5-di((E)-benzylidene)piperidin-4-one (**2a**)

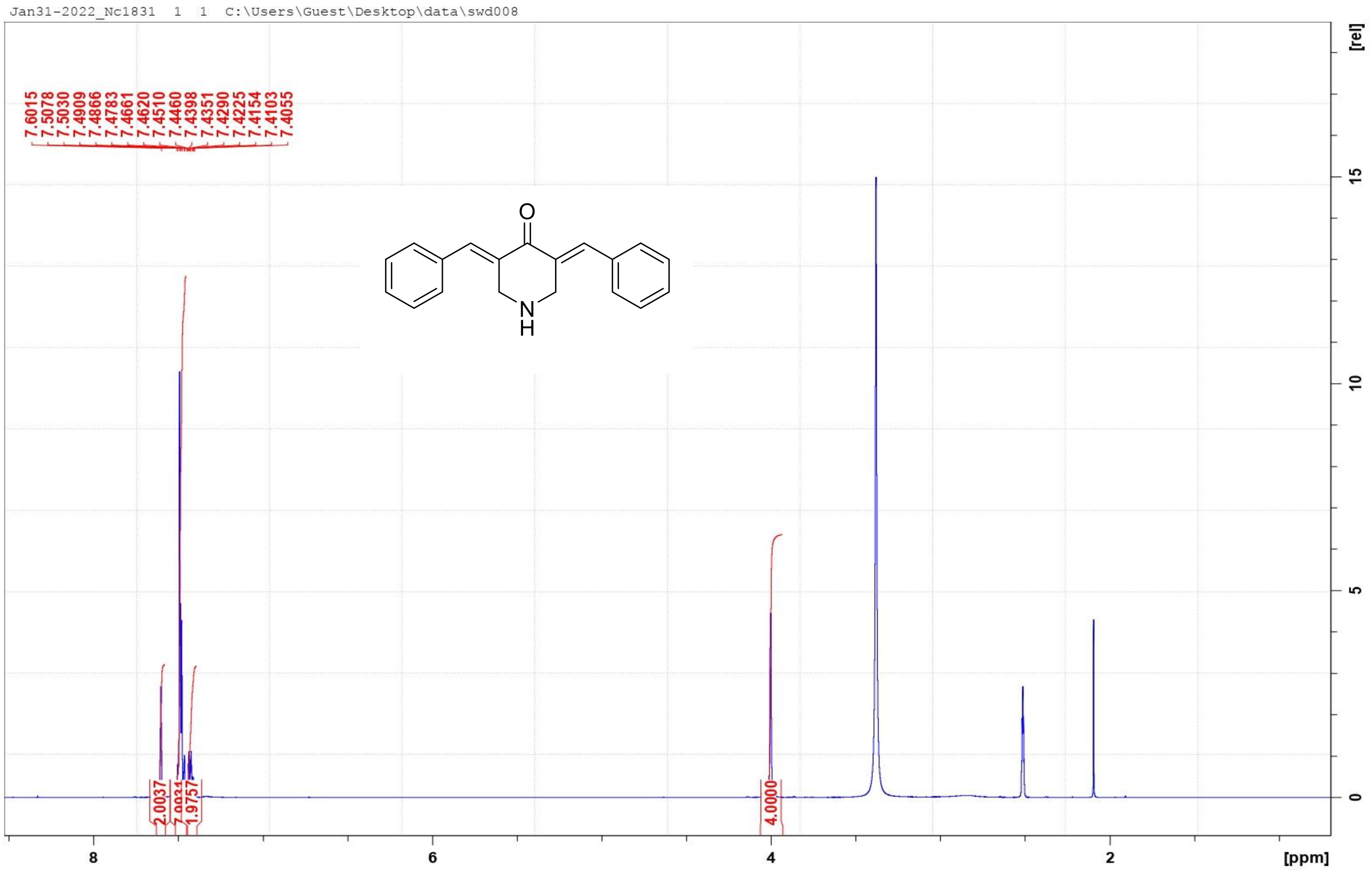

^{13}C NMR ($(CD_3)_2SO$) spectrum of 3,5-di((E)-benzylidene)piperidin-4-one (**2a**)

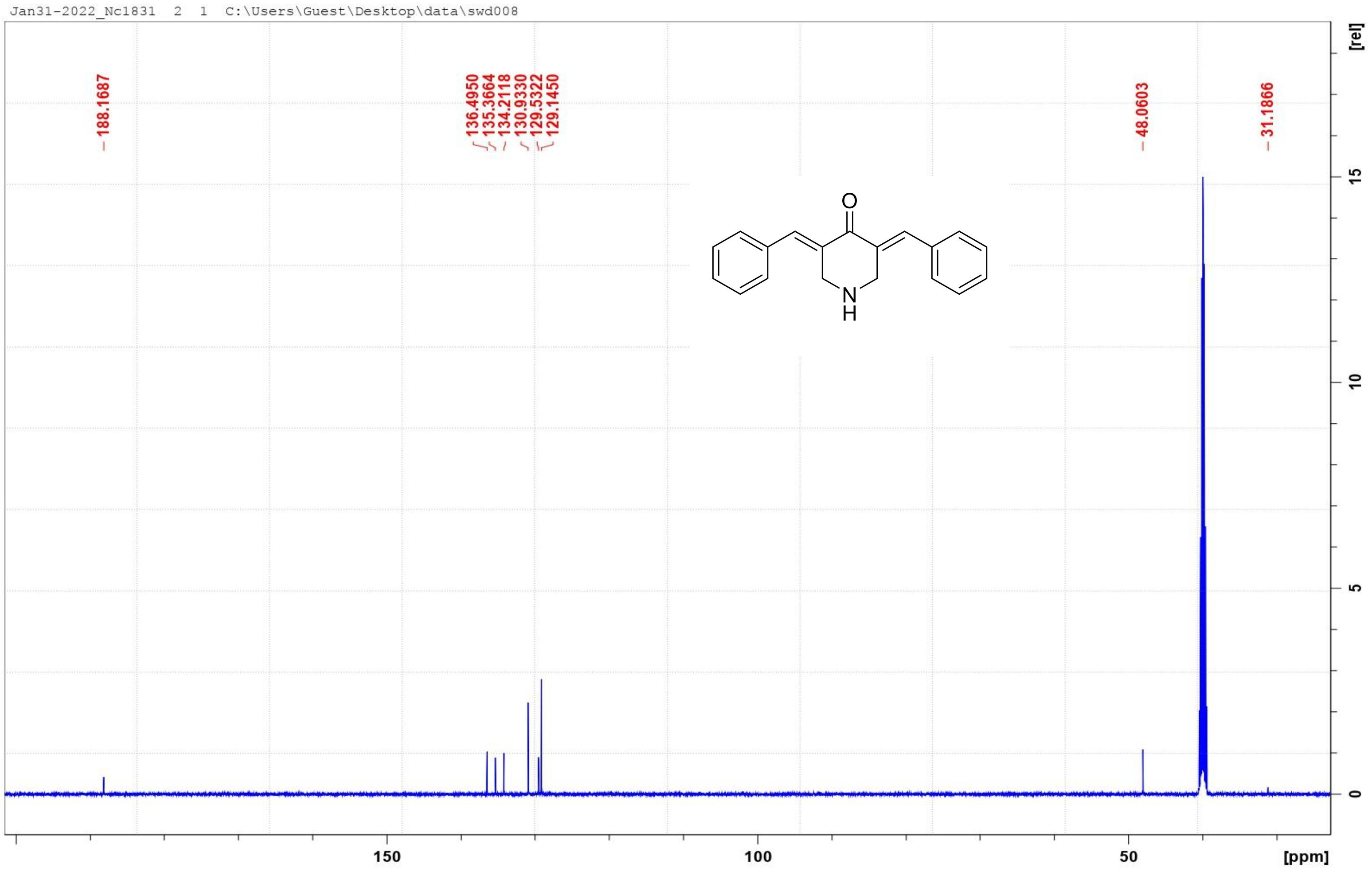

Mass spectrum of 3,5-di((E)-benzylidene)piperidin-4-one (**2a**)

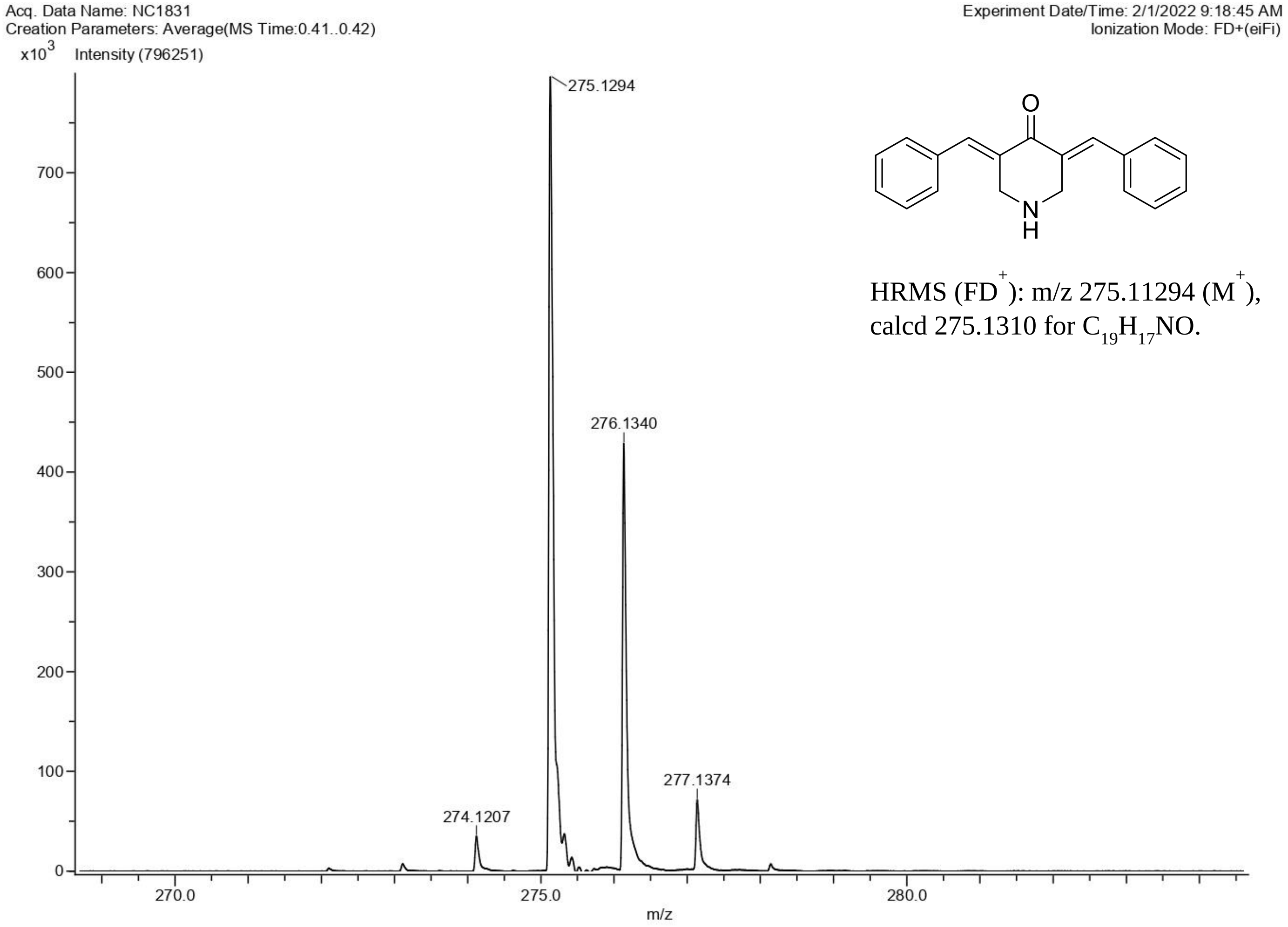

HRMS (FD^+): m/z 275.11294 (M^+), calcd 275.1310 for $C_{19}H_{17}NO$.

^{1}H NMR ($(CD_3)_2SO$) of 1-acetyl-3,5-di((E)-benzylidene)piperidin-4-one (**2b**)

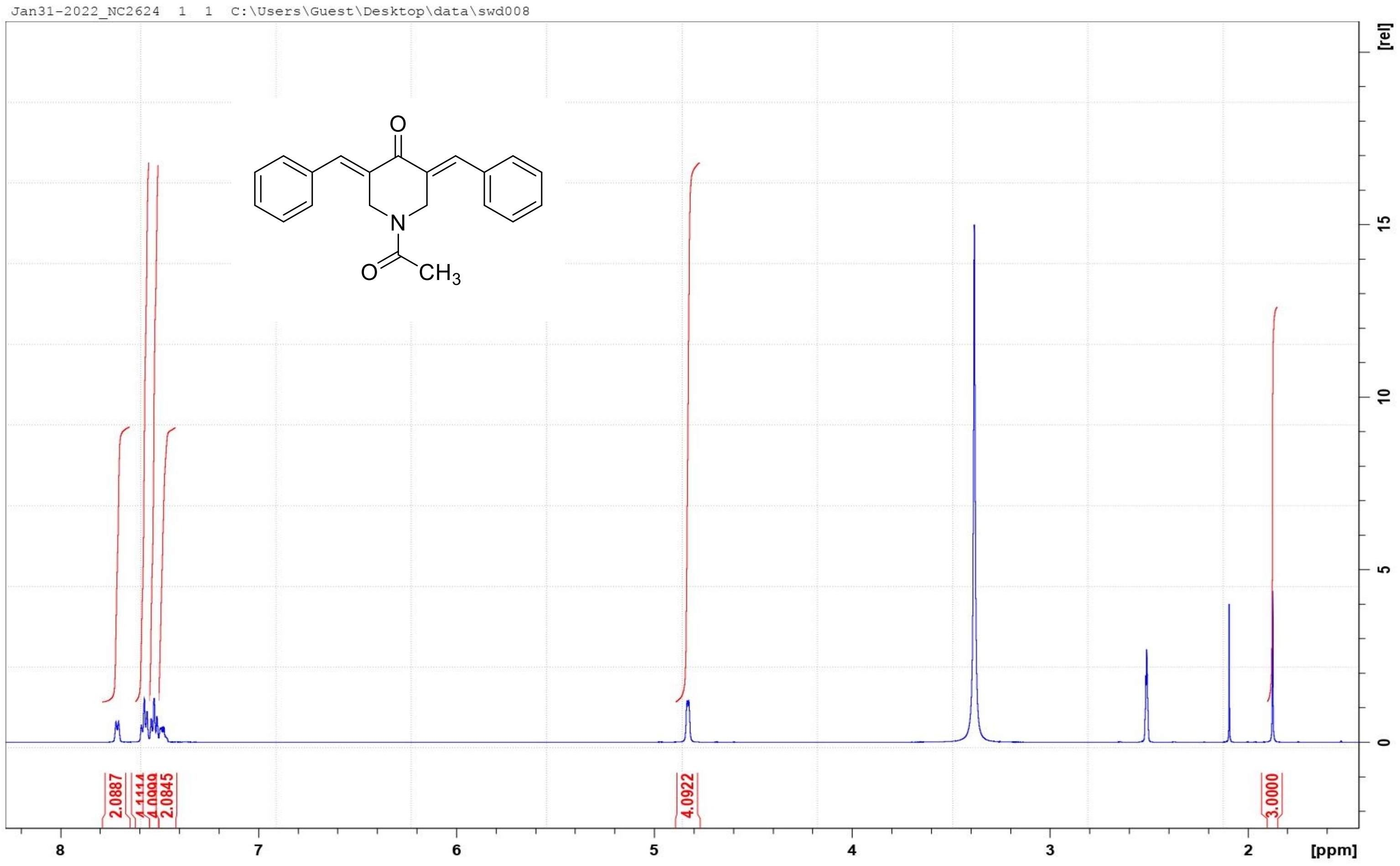

^{13}C NMR ($(CD_3)_2SO$) spectrum of 1-acetyl-3,5-di((E)-benzylidene)piperidin-4-one (**2b**)

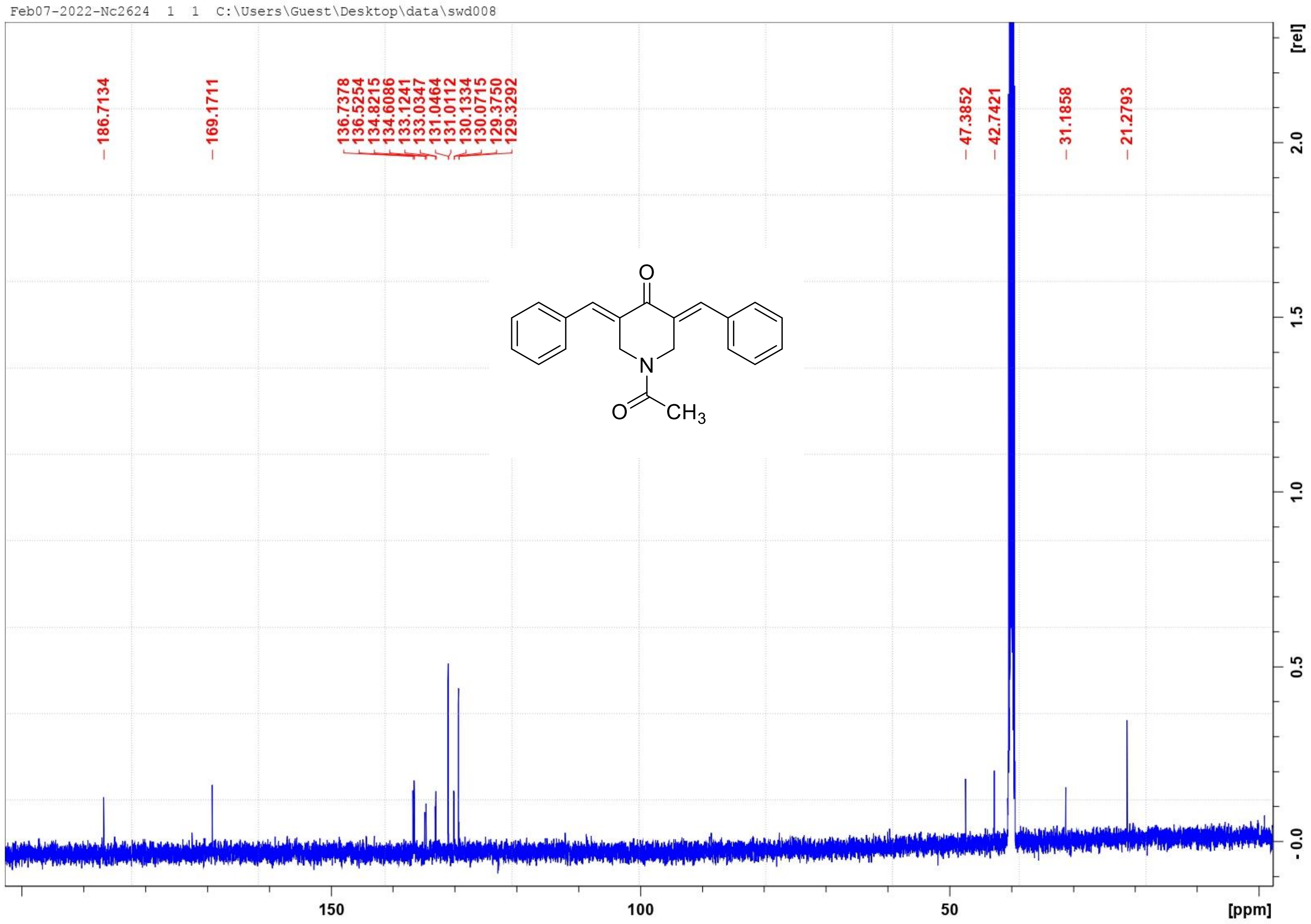

Mass spectrum of 1-acetyl-3,5-di((E)-benzylidene)piperidin-4-one (**2b**)

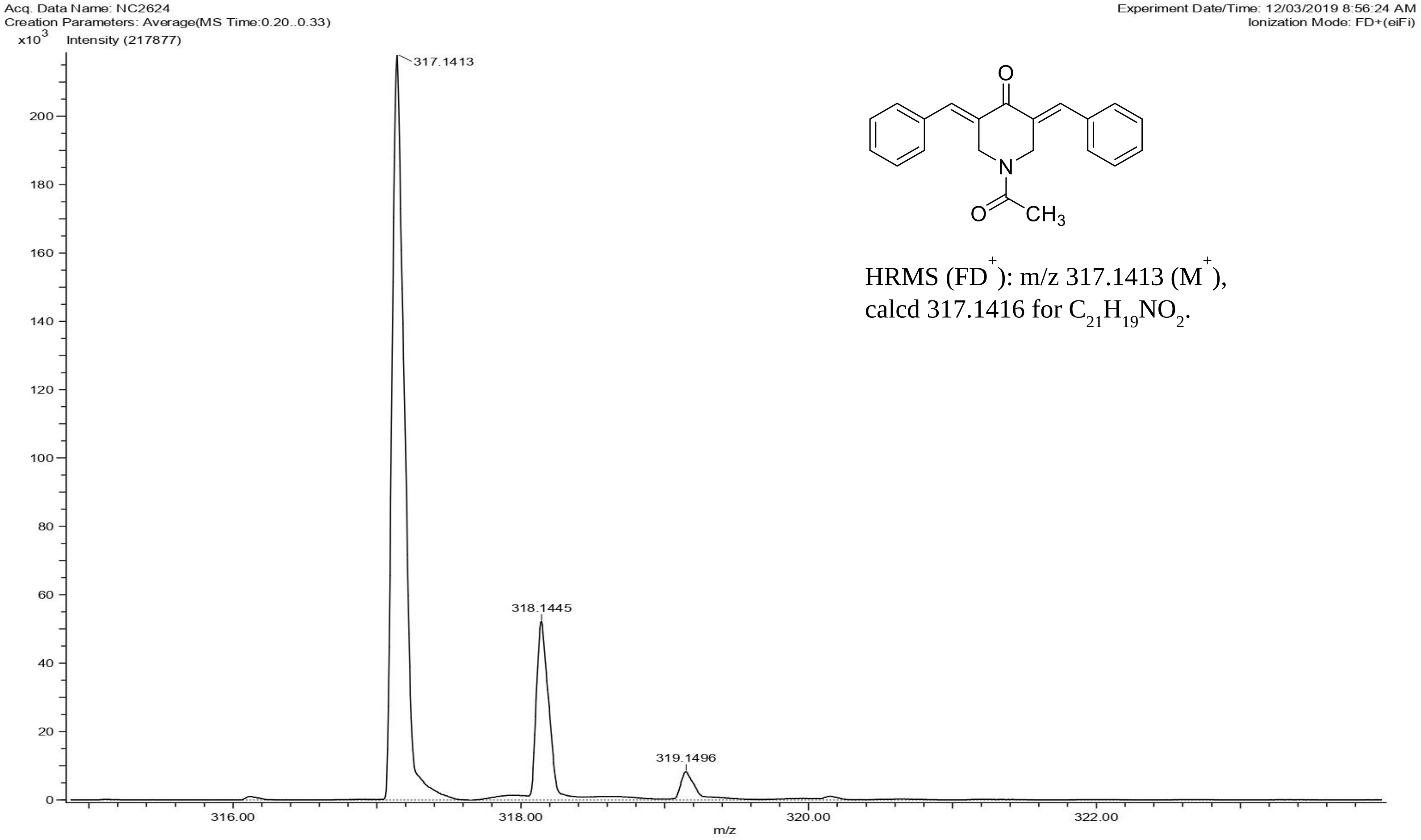

HRMS (FD^+): m/z 317.1413 (M^+), calcd 317.1416 for $C_{21}H_{19}NO_2$.
